# Genetic requirements for *Staphylococcus aureus* nitric oxide resistance and virulence

**DOI:** 10.1371/journal.ppat.1006907

**Published:** 2018-03-19

**Authors:** Melinda R. Grosser, Elyse Paluscio, Lance R. Thurlow, Marcus M. Dillon, Vaughn S. Cooper, Thomas H. Kawula, Anthony R. Richardson

**Affiliations:** 1 Department of Microbiology and Immunology University of North Carolina at Chapel Hill Chapel Hill, North Carolina, United States of America; 2 Department of Microbiology and Molecular Genetics University of Pittsburgh, Pittsburgh, Pennsylvania, United States of America; 3 Paul G. Allen School for Global Animal Health Washington State University, Pullman, Washington, United States of America; University of Tubingen, GERMANY

## Abstract

*Staphylococcus aureus* exhibits many defenses against host innate immunity, including the ability to replicate in the presence of nitric oxide (NO·). *S*. *aureus* NO· resistance is a complex trait and hinges on the ability of this pathogen to metabolically adapt to the presence of NO·. Here, we employed deep sequencing of transposon junctions (Tn-Seq) in a library generated in USA300 LAC to define the complete set of genes required for *S*. *aureus* NO· resistance. We compared the list of NO·-resistance genes to the set of genes required for LAC to persist within murine skin infections (SSTIs). In total, we identified 168 genes that were essential for full NO· resistance, of which 49 were also required for *S*. *aureus* to persist within SSTIs. Many of these NO·-resistance genes were previously demonstrated to be required for growth in the presence of this immune radical. However, newly defined genes, including those encoding SodA, MntABC, RpoZ, proteins involved with Fe-S-cluster repair/homeostasis, UvrABC, thioredoxin-like proteins and the F_1_F_0_ ATPase, have not been previously reported to contribute to *S*. *aureus* NO· resistance. The most striking finding was that loss of any genes encoding components of the F_1_F_0_ ATPase resulted in mutants unable to grow in the presence of NO· or any other condition that inhibits cellular respiration. In addition, these mutants were highly attenuated in murine SSTIs. We show that in *S*. *aureus*, the F_1_F_0_ ATPase operates in the ATP-hydrolysis mode to extrude protons and contribute to proton-motive force. Loss of efficient proton extrusion in the Δ*atpG* mutant results in an acidified cytosol. While this acidity is tolerated by respiring cells, enzymes required for fermentation cannot operate efficiently at pH ≤ 7.0 and the Δ*atpG* mutant cannot thrive. Thus, *S*. *aureus* NO· resistance requires a mildly alkaline cytosol, a condition that cannot be achieved without an active F_1_F_0_ ATPase enzyme complex.

## Author summary

The human pathogen *Staphylococcus aureus* is remarkably resistant to many facets of the host immune response, including the antibacterial radical nitric oxide (NO·). The mechanism underlying this resistance is complex and comprises many gene products. Here we employ an approach that involves transposon mutagenesis coupled to next-generation sequencing (known as Tn-Seq) to identify the complete set of genes required for *S*. *aureus* NO· resistance and virulence. While we identified many previously reported NO·-resistance determinants, new gene products were discovered from this untargeted approach. Specifically, we identified the F_1_F_0_ ATPase as being essential during NO· stress and virulence yet dispensable under normal culture conditions. The reason for this conditional fitness contribution stems from the fact that under fermentative conditions, the F_1_F_0_ ATPase functions in the ATP hydrolysis mode, effectively extruding protons and raising the intracellular pH above 8.0. This happens to be the optimal pH for many fermentation enzymes. Without the F_1_F_0_ ATPase, proton extrusion is limited and the intracellular pH remains too low for efficient fermentation to continue. Thus, during infection when *S*. *aureus* must ferment due to the nature of inflamed tissue, the F_1_F_0_ ATPase becomes an essential enzyme complex and a valid target for the development of new antimicrobials.

## Introduction

*Staphylococcus aureus* is a highly invasive human pathogen that is responsible for significant morbidity each year[1]. Treatment of *S*. *aureus* infections has become increasingly difficult due to the propensity of *S*. *aureus* to quickly evolve antibiotic resistance. Methicillin resistant *S*. *aureus* (MRSA) and multidrug resistant MRSA clones are now prevalent throughout the world[2]. Although historically a nosocomial pathogen, in recent decades otherwise healthy individuals have begun to contract MRSA outside of hospital settings[3]. These community-acquired MRSA (CA-MRSA) strains are typically characterized as hypervirulent and most frequently cause skin and soft tissue infections (SSTIs), although infections often progress to more invasive and systemic disease[4].

Understanding the factors contributing to the persistence and invasiveness of CA-MRSA is of paramount importance to limiting its current spread through both community and hospital settings. A major factor contributing to *S*. *aureus* persistence within mammalian hosts is resistance to the antimicrobial radical nitric oxide (NO·), a membrane permeable gas that is produced by activated phagocytes in response to bacterial infection[5]. Production of NO· is important for limiting bacterial proliferation in multiple infection models, so the ability to continue growth in the presence of this broad-spectrum antimicrobial confers *S*. *aureus* with a major pathogenic advantage. *S*. *aureus* NO· resistance is a highly unique trait, as even closely related Staphylococci such as *Staphylococcus epidermidis*, *S*. *saprophyticus* and *S*. *haemolyticus* are sensitive to NO·[6,7]. Additionally, NO· resistance is important for *S*. *aureus* persistence in murine infection models because deletion of genes required for NO· resistance attenuates *S*. *aureus* virulence[6,8,9].

Exposure to NO· results in a myriad of consequences within a bacterial cell. NO· and its derivatives target metal centers of bacterial enzymes (heme iron, iron-sulfur clusters, and other transition metal cofactors) and protein thiols[10,11]. At high NO· concentrations, the reversible binding of NO· to cytochrome heme iron results in respiration inhibition[12]. NO· and its derivatives can also cause DNA damage, lipid peroxidation, and nitration of tyrosine residues[13]. *S*. *aureus* must therefore employ a diverse array of strategies to cope with these multifaceted effects.

We have previously characterized several of the strategies employed by *S*. *aureus* to resist the effects of NO·. The induction of a unique L-lactate dehydrogenase enzyme, Ldh1, in response to NO· exposure is important for the ability of *S*. *aureus* to balance redox when respiration is inhibited by NO·[12]. The NO·-mediated activation of the two-component system SrrAB when respiration is limited by NO· results in induction of a flavohemoprotein, Hmp, that detoxifies NO· to nitrate[8]. SrrAB also induces increased expression of two terminal oxidases, Qox and Cyd, which helps to overcome the inhibitory effects of NO· on respiration[14,15]. However, with the exception of Ldh1, these responses to NO· stress are not unique to *S*. *aureus* and cannot fully explain its resistance. *Bacillus subtilis* has both a two-component system homologous to SrrAB and an NO·-inducible Hmp homologue, yet is highly sensitive to this immune radical[16]. Therefore, we are still lacking a significant understanding of what makes the *S*. *aureus* response to NO· so unique and effective.

Several genome-wide transposon-based screens have been performed to date with the goal of identifying genes important for *S*. *aureus* pathogenesis in multiple infection models, including murine bacteremia, skin abscess, osteomyelitis, rabbit endocarditis, and nematode infection[17–21]. Many of these studies used signature-tagged mutagenesis and thus employed a small and highly limited pool of transposon mutants for screening[17,18]. Other studies used MSSA strains of *S*. *aureus* with significant virulence differences relative to CA-MRSA isolates[19,20]. A recent Tn-Seq screen using the MSSA strain HG003 examined Tn-mutant fitness after 24 and 48-hrs of infection in murine SSTIs and identified many genes important for persistence[20]. However, HG003 is not highly virulent in an SSTI model, and abscess bacterial burdens remained constant at around the same level as the inoculum in these experiments. Therefore, significant knowledge remains to be gained regarding genes required for fitness in *S*. *aureus* strains that are specifically known for their proclivity to proliferate within SSTIs, such as the CA-MRSA isolates.

In the current study, we report the first *in vivo* Tn-Seq screen to use a relevant CA-MRSA strain of *S*. *aureus*, USA300 LAC. We employ a genome-wide Tn-Seq screen with a highly saturated transposon library to broadly identify genes required for NO· resistance in *S*. *aureus*. After identifying genes important for NO· resistance *in vitro*, we then investigated genes important for general fitness in murine SSTIs and identified a subset of genes important for both NO· resistance *in vitro* and for persistence in murine SSTIs. Most notably, our results highlight a major role for the F_1_F_0_ ATPase during both NO· stress and virulence in murine SSTIs. This phenomenon may be generalizable across bacterial species as the F_1_F_0_ ATPase was recently reported as being essential to *Listeria monocytogenes* when cultured anaerobically[22]. Thus, given the non-respiratory nature of inflamed tissue environments (hypoxia, iron-limitation and high levels of immune radicals), the F_1_F_0_ ATPase may be required for the full virulence of a variety of pathogenic microorganisms, underscoring the utility of targeted antimicrobials that inhibit this enzyme complex[23].

## Results

### Creation of a high-density transposon library in *S*. *aureus* LAC

**Fig 1:**
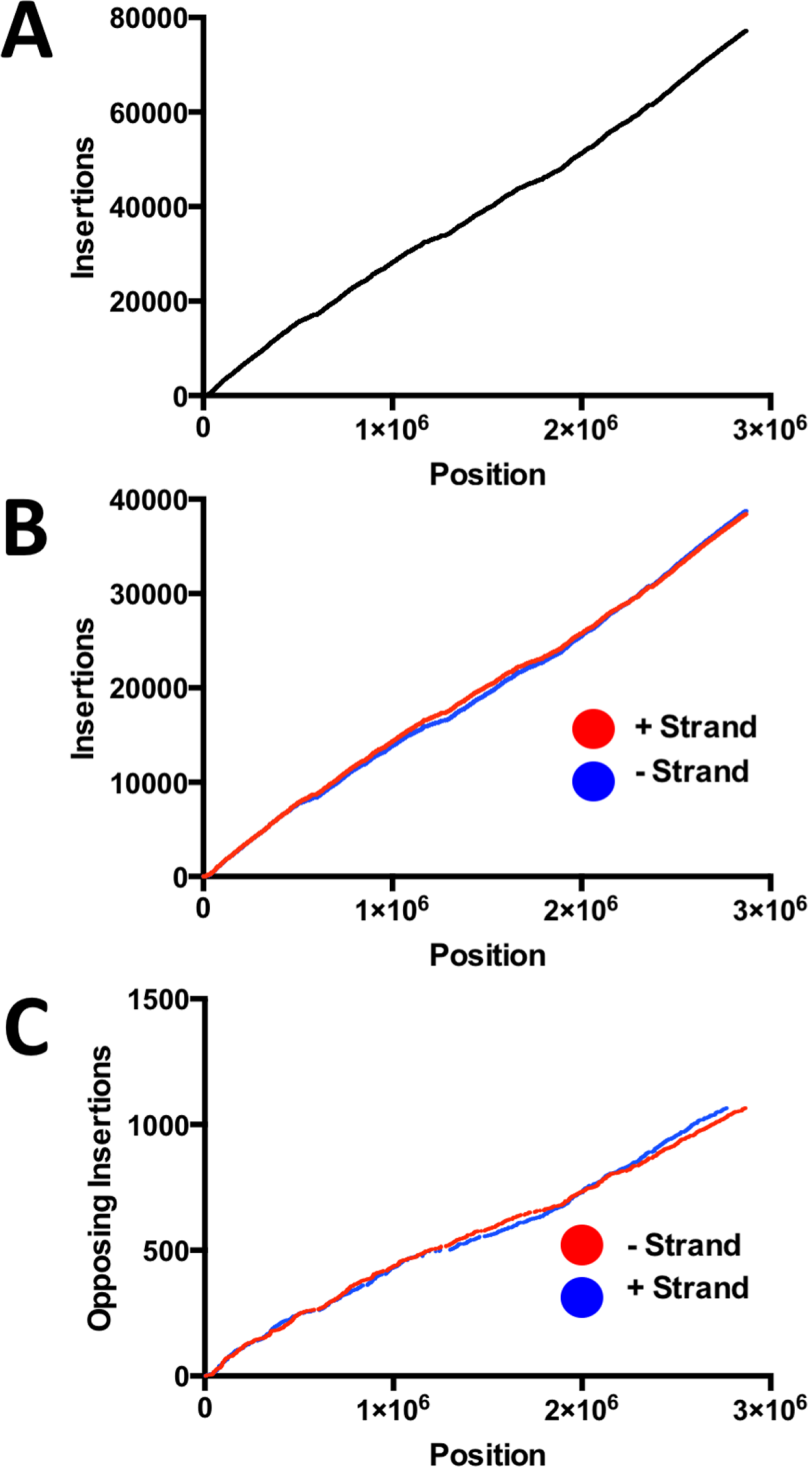
Distribution of Tn-insertions along the *S*. *aureus* genome. **A.** Insertions appear uniform all along the genome with no obvious gaps or hot spots. **B.** Distribution of insertions on each strand reveal no apparent strand bias. **C.** 2,132 Tn insertions were at identical TA dinucleotides but on opposite strands. The 1066 sites appear to be uniformly distributed across the entire genome.

Previous genome-wide screening efforts to identify essential genes in *S*. *aureus* have used MSSA strains including Newman, RN4220, RN6390, SH1000 and HG003[17–21,24]. Because our primary interest was to identify genes required for both NO· resistance and persistence in SSTIs, we wanted to select a MRSA strain commonly associated with SSTIs in the current CA-MRSA pandemic. Therefore, we chose to generate a high-density transposon library in *S*. *aureus* LAC, belonging to the dominant CA-MRSA PFGE subtype, USA300. We used the transposon *bursa aurealis*, a mariner-based transposon that inserts randomly throughout the *S*. *aureus* genome into TA dinucleotides[21,25]. The Himar1 transposase-containing plasmid pFA545 that has previously been used with *bursa aurealis* in *S*. *aureus* did not successfully result in transposition, so we modified this plasmid by replacing the original transposase allele and its xylose-inducible promoter with a new copy of the transposase allele fused to the constitutive *lgt* promoter. This plasmid mediated efficient transposition and was used for creation of a saturated *S*. *aureus* LAC transposon library comprising 77,161 independent Tn insertions (Fig 1A). To verify that constitutive expression of *tnp* was mediating only one transposition event per genome, we performed a Southern blot with a Tn-specific probe on *Cla*I digests of DNA from sixteen individually isolated Tn-mutants. We observed single bands of variable sizes for each mutant, indicating that transposition was occurring randomly and only once per genome for each of the 16 mutants (S1 Fig). Moreover, the density of coverage was very high with a median distance between two neighboring Tn insertion sites at 16 bp (S2 Fig). The median distance metric is not subject to the effects of essential DNA segments that significantly elevate the average distance between neighboring insertions. The non-biased nature and high density of the Tn insertions is further reflected by a lack of obvious strand bias (Fig 1B) including within the 2,132 Tn insertions that were at the identical site in the genome, but inserted into opposing DNA strands (Fig 1C).

**Fig 2:**
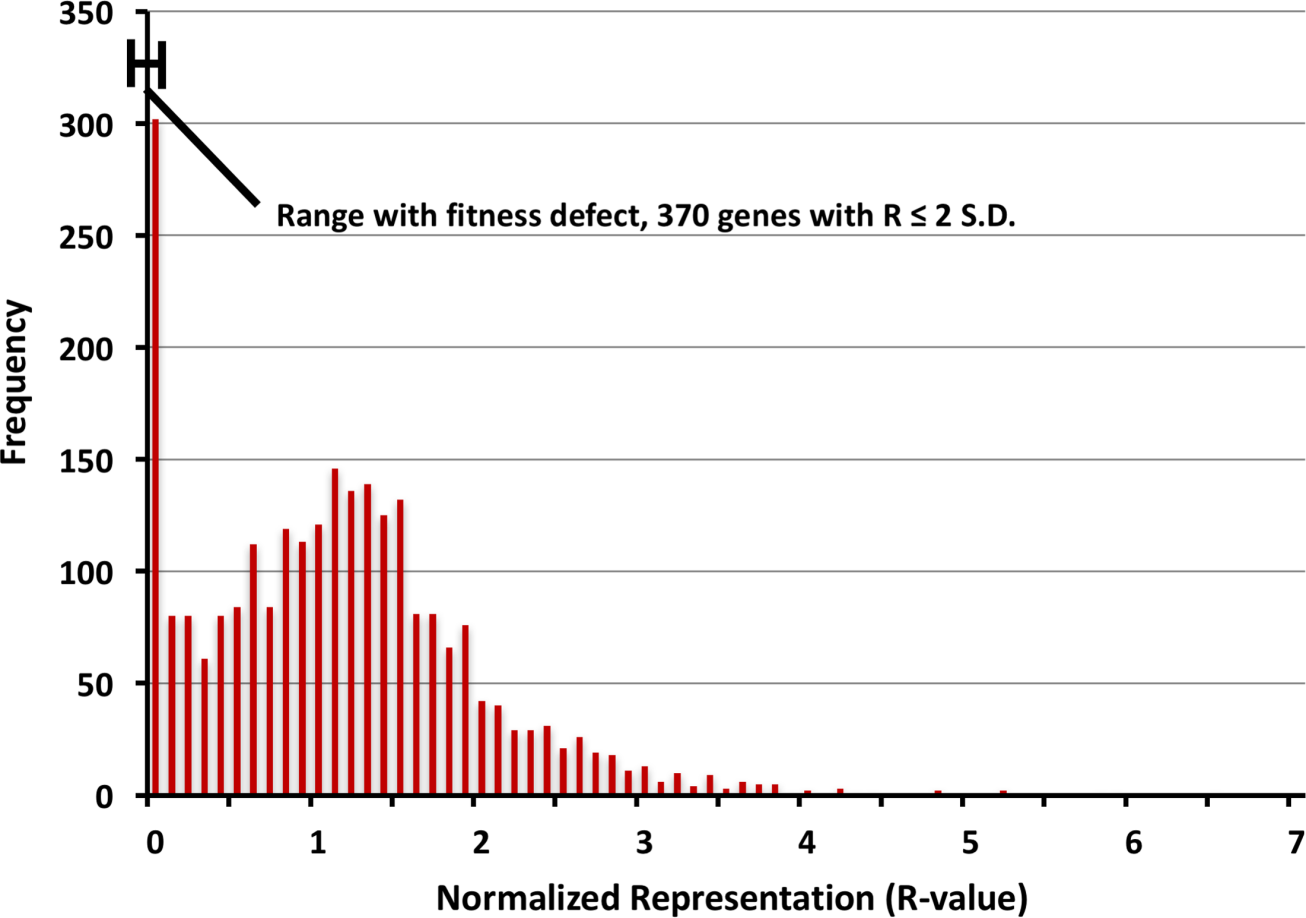
Biphasic distribution of representation values (R) for all genes in *S*. *aureus*. R-values below 3 SD of the log transformed mean are considered essential whereas R-values between 2 and 3 SD below the mean are considered to be required for full fitness.

To calculate the relative fitness contribution of genes we generated representation (R) values based on both the density of transposon insertions and the number of reads, where R = (median read count) x (insertion density) for each gene (see Materials and Methods). For each run, individual R-values were all normalized to the median R-value of the run to correct for variation in read numbers. Thus, the typical R-value distribution is biphasic with a peak at or near 1 for genes with no significant contribution to fitness within a given environment and a peak at 0 indicative of elements that are absolutely essential for survival in a given environment (Fig 2). More specifically, genes with log-transformed R-values more than three standard deviations (SD) below the mean R value were designated as being essential, while those with log-transformed R-values between 2 and 3 SD below the mean were designated as genes required for full fitness. By this calculation, we found 370 genes to be essential/required in our input library. Our findings are consistent with previous reports[20], with 88% of the essential genes in our dataset being previously designated as essential despite the fact that the two libraries were generated in different strain backgrounds (HG003 versus LAC). Furthermore, comparing essential gene lists between this study and those by Valentino *et al*. and Chaudhuri *et al*. finds that the majority of genes (288) were commonly identified between all three studies (S3 Fig and S1 Table) [20,24]. R values for all genes in the library under all conditions tested are listed in S2 Table.

### Identification of novel NO·-resistance determinants in *S*. *aureus*

Given the extensive overlap between our list of essential genes with those previously reported, we chose not to validate our essential genes. Rather, we focused on applying Tn-Seq to identify undiscovered NO·-resistance determinants in *S*. *aureus* as well as genes required for persistence within 3-day and 7-day murine skin abscesses. To generate inocula for further selection experiments, we grew library aliquots for 10-hrs overnight in TSB 5mg/ml glucose. We quantified transposon insertions from two different 10-hr overnight cultures to establish the composition of our inoculation culture. We also performed a technical replicate of library preparation for Illumina sequencing on one overnight culture to establish the reproducibility of our method. We performed regression analyses of the insertion densities (# of actual insertions/ # of possible insertions for each gene) between replicates and found a high degree of reproducibility between both technical and biological replicates, most with Pearson coefficients of ~0.9 (S4 Fig).

**Fig 3:**
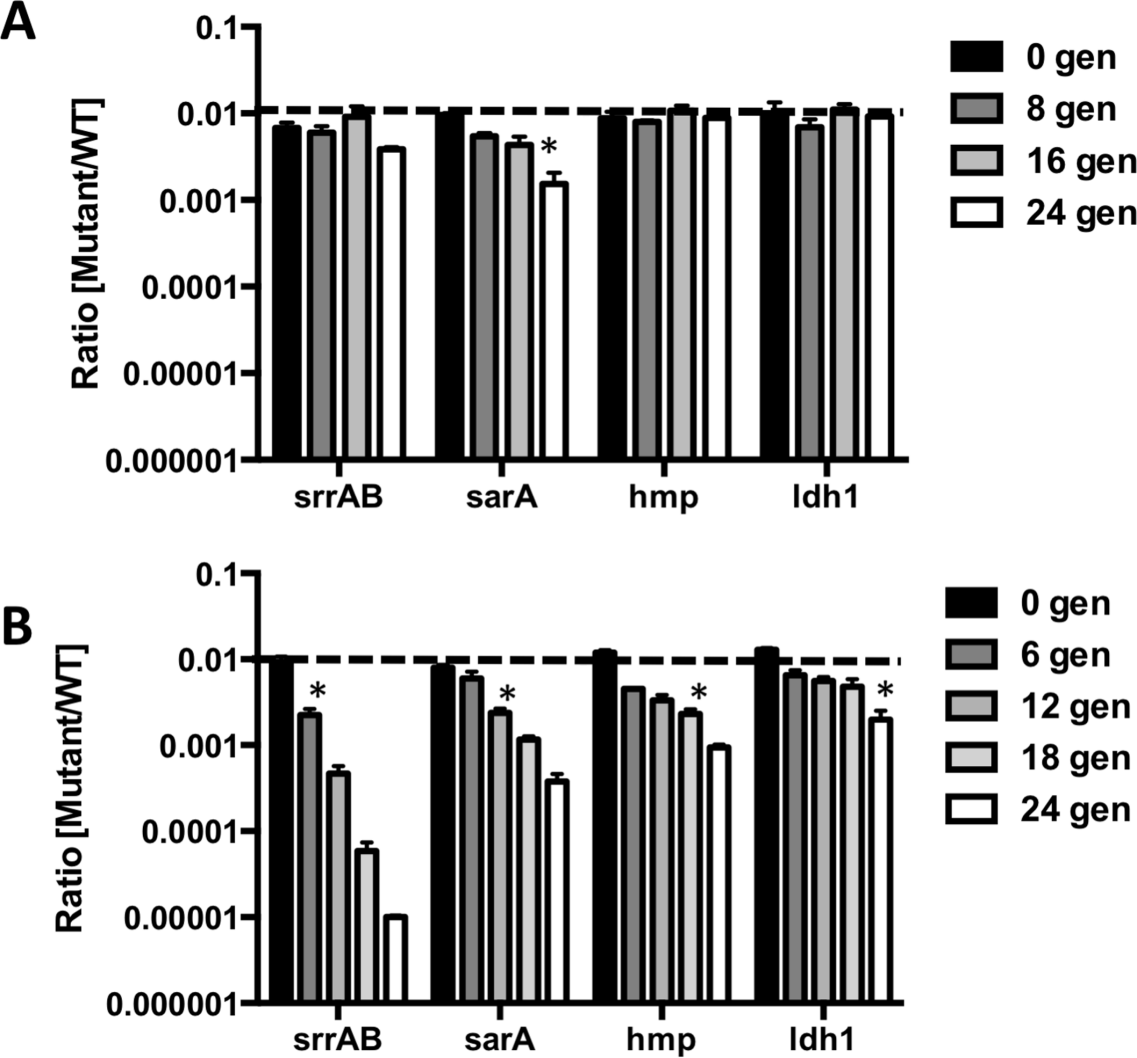
Optimization of an assay to select for mutant fitness during NO· stress. Known NO· sensitive mutants in *S*. *aureus* LAC were mixed with WT at a ratio of 1:100. Mixed cultures were serially passaged either **A.** aerobically or **B.** with 5mM DETA/NO. Every 12-hrs (for NO· exposed) or 5-hrs (for aerobic), cultures were diluted 1:100 into fresh media (with or without DETA/NO) and were plated on selective media for cfu enumeration. Asterisks indicate the generation at which the mutant becomes significantly underrepresented from the initial 1:100 ratio (n = 3 for each mutant, Student’s t-test).

Before performing Tn-Seq to identify NO· sensitive Tn-mutants, we first validated an assay for fitness selection in the presence of NO·. We mixed cultures of WT LAC and several known NO· sensitive mutants: Δ*srrAB*, Δ*hmp*, Δ*ldh1*, and Δ*sarA*. These mutants were selected because they exhibit a range in severity of NO· sensitivity and are sensitive to NO· via a variety of mechanisms[8,12,14]. We mixed each mutant with WT in a 1:100 ratio to simulate the fact that in the saturated Tn-library, the majority of bacterial cells will not have fitness defects in the presence of NO·. These mixed cultures were serial passaged aerobically with or without NO· for 24 generations. Over time, NO· sensitive mutants were out-competed by WT in the presence of NO· but not significantly when grown aerobically (Fig 3) with the only exception being Δ*sarA*. However, the underrepresentation of the Δ*sarA* mutant passaged for 24 generations under NO· stress was significantly exacerbated when compared to aerobically passaged cultures (Fig 3). Thus, 24 generations under constant NO· stress is sufficient to select against mutants with varying degrees of NO·-sensitivity.

We next serially passaged the Tn-library aerobically either with or without NO· as described above for 24 generations, performing two biological replicates for each condition. We averaged the R-values from the two biological replicates and, similarly to methods outlined above, we determined genes essential for serial passage in aerobic cultures (genes with R-values ≥ 3 SD below the mean normalized R-value). These genes were removed from further analyses since they are required for aerobic growth in the absence of NO·. We then computed the ratios of R-values of remaining genes from NO·-passaged cells to those of aerobically grown cells for each gene. There were a total of 41 genes that were essential (≥ 3 SD below mean NO·:aerobic ratio) for NO· resistance and 127 genes with significant fitness contributions during NO· stress (NO·:aerobic ratios between 2 and 3 SD below the mean, S3 Table). These included many previously characterized NO· resistance determinants such as *ldh*1 (SAUSA300_0235), *hmp* (SAUSA300_0234), *pyk* (SAUSA300_1644), *ccpA* (SAUSA300_1682), *codY* (SAUSA300_1148), *nfu* (SAUSA300_0839), *srrAB* (SAUSA300_1442/1) and *qoxABD* (SAUSA300_0963/2/0) [8,9,12,14,26,27]. However, additional genes not previously known to contribute to NO· resistance in *S*. *aureus* were among the essential/required list including genes encoding the F_1_F_0_ ATPase (SAUSA300_2057 through SAUSA300_2064), SodA (SAUSA300_1513), MntABC (SAUSA300_0618, SAUSA300_0619 SAUSA300_0620), UvrA (SAUSA300_0742), UvrC (SAUSA300_1045), MprF (SAUSA300_1255) and RpoZ (SAUSA300_1103). Other genes important for fitness specifically during NO· stress included genes associated with carbohydrate utilization (*ptsI*/SAUSA300_0984 and *gpmI/*SAUSA300_0759), proteases (*clpC*/SAUSA300_0510), putatively involved in iron-sulfur cluster homeostasis (SAUSA300_1248), thioredoxin-like proteins (SAUSA300_0903), transcriptional regulators (*ctsR*/SAUSA300_0507, *sarT*/SAUSA300_2437 and the two-component system *bceRS*/SAUSA300_0645/6) and SigB regulation (*rsbU/*SAUSA300_2025 and *rsbW*/SAUSA300_2023). There were an additional 11 genes that were overrepresented by 3 SD in NO·-stressed cultures and 38 genes between 2 SD and 3 SD overrepresented. These mainly consist of hypothetical proteins and genes encoding enzymes involved in nucleotide and amino acid metabolism (*e*.*g*. *apt/*SAUSA300_1591, *carA*/SAUSA300_1095, *aroK*/SAUSA300_1499, *aroD/*SAUSA300_0787, *gudB*/SAUSA300_0861, *thrC*/SAUSA300_1227 and *leuD*/SAUSA300_2013). It is possible that the slowed growth of these mutants (many were significantly impaired in serial aerobic cultivation) provides an advantage during NO· stress.

### Validation of NO· sensitive mutants

**Fig 4:**
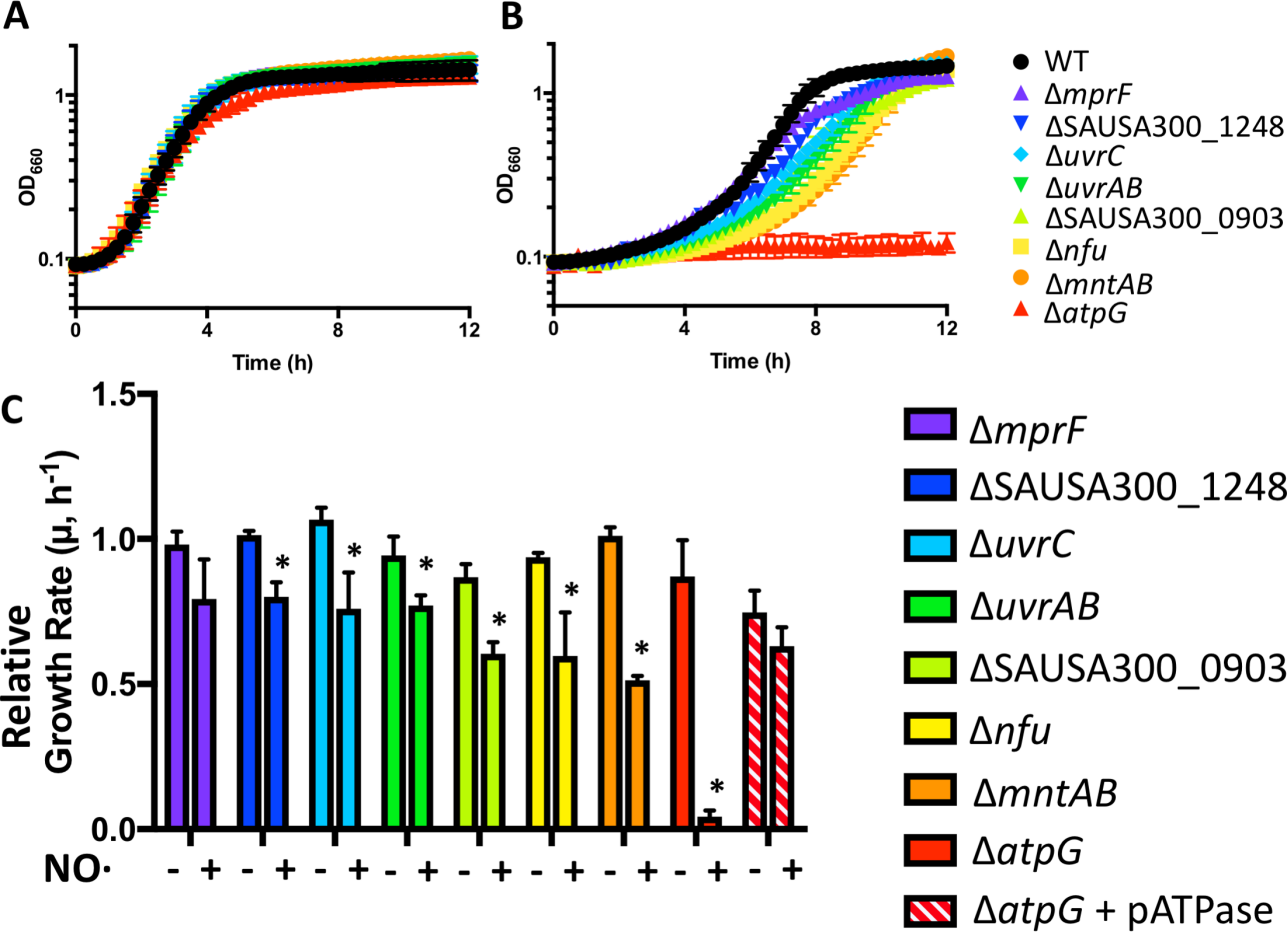
NO· sensitive mutants identified by Tn-Seq are confirmed with clean deletion mutants. Representative growth curves are shown of *S*. *aureus* LAC WT and mutants grown in TSB 5g/L glucose either **A.** aerobically or **B.** with 10mM DETA/NO added at the time of inoculum (n = 3). **C.** Growth rates of mutants and a complemented Δ*atpG* relative to that of WT with/without NO· exposure at inoculum (10 mM DETA/NO).

To validate the results of Tn-Seq identification of genes required for NO· resistance, we created clean deletion mutants of five genes or operons newly identified as required for growth during NO· stress: *atpG* (F_1_F_0_ ATPase), *uvrAB* and *uvrC* (nucleotide excision repair), *mntABC* (manganese ABC transporter), and *mprF* (antimicrobial peptide resistance factor). Additionally, we tested mariner Tn-mutants from the Nebraska Library including iron-sulfur cluster assembly genes *nfu* and SAUSA300_1248 as well as a thioredoxin-like protein, SAUSA300_0903. With the exception of Δ*mprF*, all other mutants exhibited longer lag times and slower growth rates than WT in the presence of NO· but not during aerobic growth, confirming their identification as important for NO· resistance (Fig 4). The lack of NO· sensitivity observed for Δ*mprF* could be due to the shorter NO· exposure time in the growth curve, or to differences in growth conditions between a 96-well plate (where growth curves were performed) and a shaking 5 ml culture in a test tube (where selection experiments were performed).

### Genes required for fitness in murine SSTIs

To identify genes important for fitness in murine SSTIs, we infected mice subcutaneously with 10^7^ cfu of the Tn-Library and harvested bacterial DNA from skin abscesses on days 3 and 7 post infection. Abscesses from two mice were pooled at each time point, and two biological replicates were performed (for a total of four mice at each time point). We averaged R-values between biological replicates at each time point and compared them to the R-value of the 10-hr overnight culture as this was our input library for infection. Importantly, the reproducibility between biological replicates of animal infection was very high (S4 Fig, Pearson coefficients of 0.9 and 0.7 for days 3 and 7 respectively), indicating that consistent selective pressures were encountered by the bacteria infecting different mice and that there are no significant bottlenecks in this infection model up until day 7.

Of the 168 genes required for fitness in the presence of NO·, 22 were also required for fitness in murine SSTIs at day 3 (i.e., Tn-insertions in these genes were underrepresented by more than 2 SD), and 49 were required at day 7 (S4 Table). These genes included the F_1_F_0_ ATPase operon, *mntABC*, *srrAB*, *ccpA*, *rpoZ*, *codY*, *qoxABCD*, *sodA*, *pyk*, *ctsR* and *rot*. We also found that an additional 144 genes were required for fitness in SSTIs at day 3 that were not required for *in vitro* NO· resistance. At day 7 there were 213 genes required for fitness that were not involved in *in vitro* NO· resistance. Many of these genes include those not analyzed in the context of NO· exposure because they exhibited significant growth defects in aerobically passaged cells, including *sarA* and genes involved in the synthesis of purines, pyrimidines, heme, aromatic amino acids and menaquinone.

As expected, genes encoding secreted virulence factors including toxins and proteases were not identified as required for fitness in SSTIs, presumably due to trans-complementation by the rest of the bacteria in the pool. An interesting group of genes required in SSTIs but not for NO· resistance included *lgt* (SAUSA300_0744) and *lspA* (SAUSA300_1089), genes associated with lipoprotein processing and attachment to diacylglycerol[28,29]. Related to this, SAUSA300_1741, a putative lipoprotein, was essential in SSTIs but not required for *in vitro* growth. Genes of the LytR-CpsA-Psr family (*msrR/*SAUSA300_1257 and SAUSA300_0958), which are thought to link wall techoic acids to peptidoglycan[30], were also required for fitness at both day 3 and day 7. These data suggest that cell envelope and cell wall modifications play a major role in persistence in SSTIs, although largely unrelated to NO· resistance. Finally, in each of the day 3 and 7 abscess output pools, mutants in each of the Agr quorum sensing genes (*agrB*/SAUSA300_1989, *agrC*/SAUSA300_1991, *agrA*/SAUSA300_1992) were consistently and significantly overrepresented.

### The Δ*atpG* mutant exhibits reduced fitness under non-respiratory conditions

**Fig 5:**
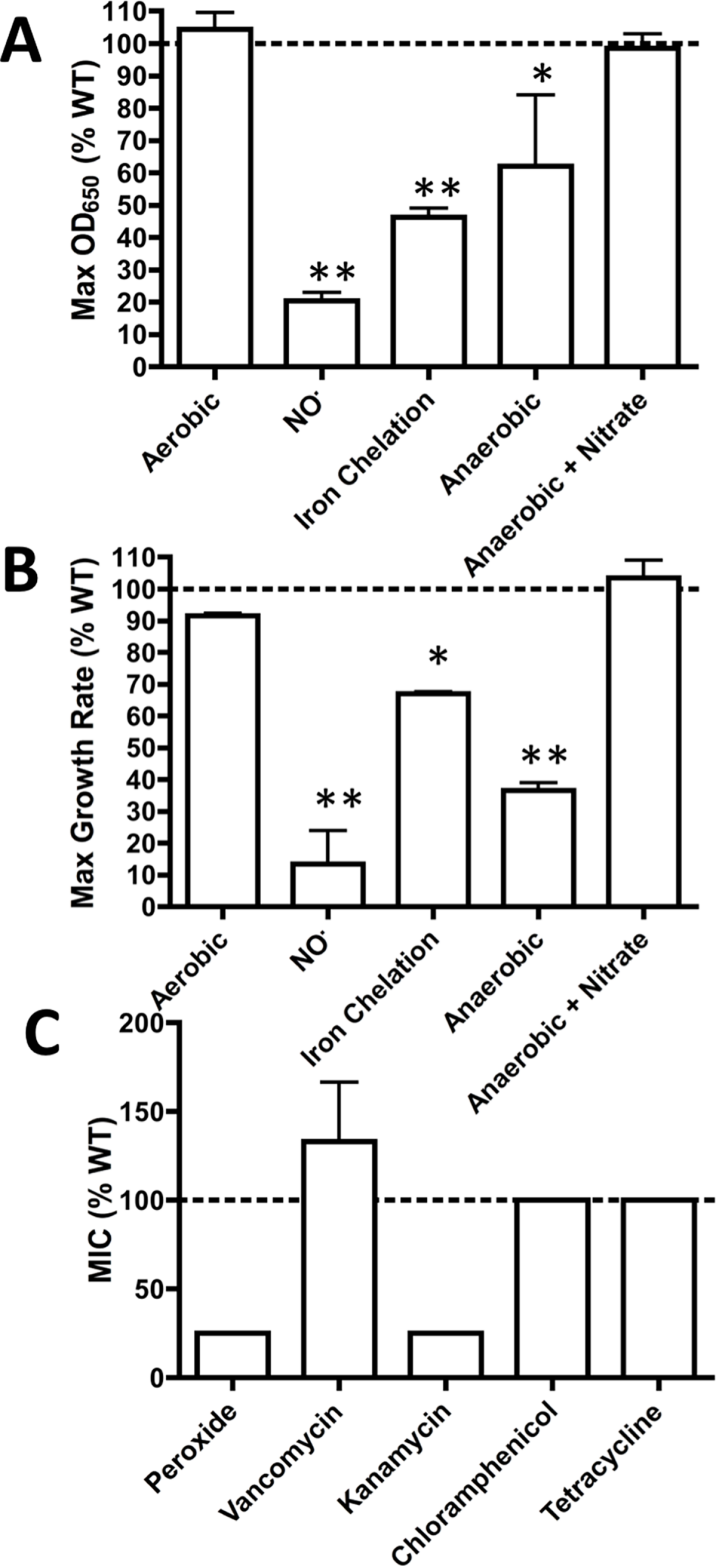
Δ*atpG* is attenuated during non-respiratory growth and is also sensitive to peroxide and kanamycin. **A.** and **B.** *S*. *aureus* LAC WT and Δ*atpG* were grown in TSB 5g/L glucose aerobically, with 10mM DETA/NO, with 2mM 2,2’-dipyridyl, anaerobically, or anaerobically with nitrate. **A.** Maximum absorbance (650nm) reached over a 24-hr growth curve and **B.** maximum growth rate reached over a 24-hr growth curve are shown for the Δ*atpG* mutant as a percentage of the WT maximums. **C.** Minimum inhibitory concentrations were measured for *S*. *aureus* WT and Δ*atpG* in TSB 5g/L glucose and are shown as a ratio to the WT MIC (n = 3).

The Δ*atpG* mutant was selected for further study because of its essentiality in the presence of NO· and in murine SSTIs both at days 3 and 7 (S3 Table and S4 Table, Fig 4, S5 Fig). Notably, the NO·-specific growth defect of the Δ*atpG* mutant could be restored by complementing with the entire *atpIBEFHAGDC* operon cloned on a medium copy vector (Fig 4). A major characteristic of high-level NO· exposure is the inhibition of respiration[12,31]. In addition, *S*. *aureus* frequently encounters hypoxia and iron limitation within inflamed tissue, two conditions that would also limit respiration. Therefore, to test whether Δ*atpG* is specifically sensitive to NO· stress or is sensitive to the general inhibition of respiration, we grew Δ*atpG* either anaerobically or in the presence of the divalent cation chelator 2’2-dipyridyl to simulate other respiration-limiting conditions encountered within a host. Δ*atpG* grew poorly compared to WT under both conditions, suggesting that its sensitivity to NO· is likely due to the general inhibition of respiration (Fig 5A and 5B). Importantly, the addition of nitrate, which *S*. *aureus* can use as an alternate electron acceptor for anaerobic respiration, rescued anaerobic growth of the Δ*atpG* mutant. These data indicate that the *S*. *aureus* F_1_F_0_ ATPase is critical for normal non-respiratory growth and is therefore likely attenuated in SSTIs due to multiple host factors that limit respiration.

We next wanted to test whether Δ*atp*G was more sensitive to general cellular stress, or whether it was specifically sensitive to respiration-limiting conditions. We found that relative to WT, Δ*atp*G was more sensitive to peroxide and kanamycin, but not to vancomycin, chloramphenicol, or tetracycline (Fig 5C). Thus, the Δ*atpG* mutant is not sensitive to cellular stress in general, but rather to specific stressors. However, these stressors are not limited to those that inhibit respiration (See Below).

### The F_1_F_0_ ATPase is required for intracellular pH homeostasis during non-respiratory growth

**Fig 6:**
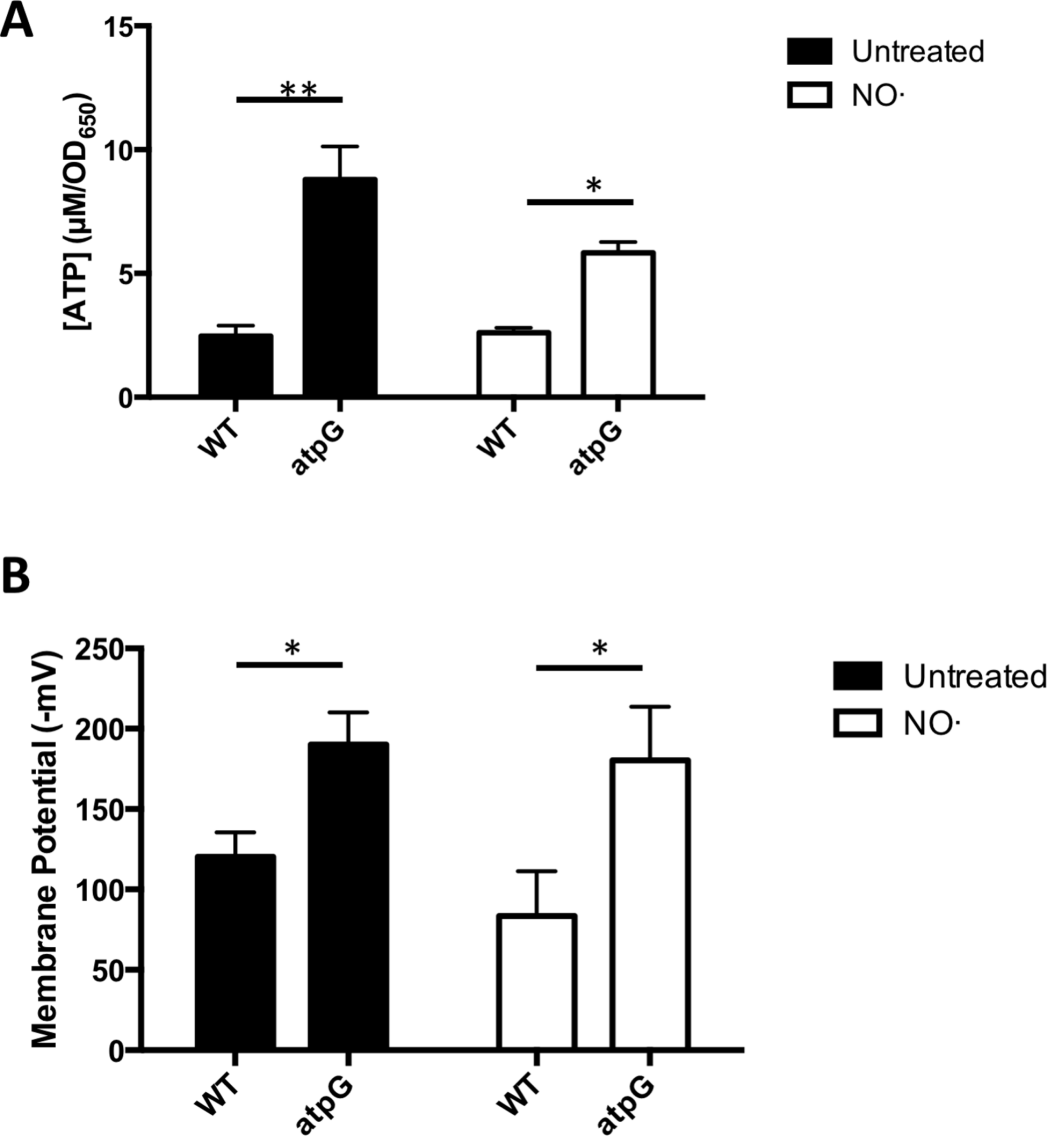
Δ*atpG* exhibits both elevated ATP levels and membrane potential both before and after NO· exposure. *S*. *aureus* WT and Δ*atpG* were grown in TSB 5g/L glucose and were exposed to NO· mix (10mM NOC12, 1mM DEANO) at OD_650_ 0.25. **A.** Prior to NO· addition and at 1-hr post addition ATP levels were determined. **B.** Prior to NO· addition and 1-hr post addition, membrane potential was quantified. (n = 2 for ATP levels, n = 3 for membrane potential). Significance was determined with two-sided Student’s t tests (*, P ≤ .05; **, P ≤ .01).

**Fig 7:**
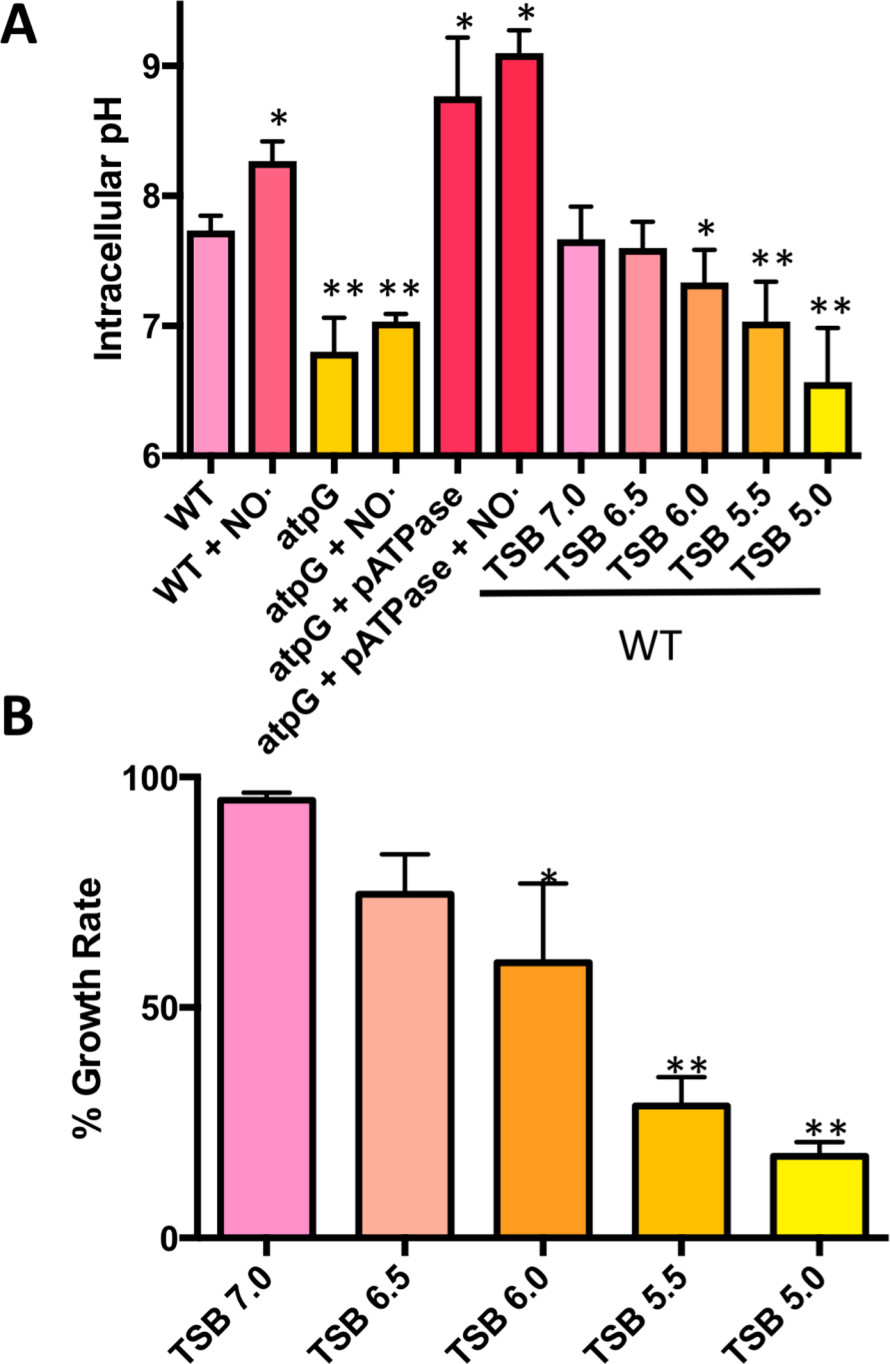
Δ*atpG* exhibits reduced intracellular pH compared to WT both in the presence/absence of NO·. **A.** *S*. *aureus* WT, Δ*atpG* and Δ*atpG* + pATPase were grown in TSB 5g/L glucose and were exposed to NO· mix (10mM NOC12, 1mM DEANO) at OD_650_ 0.25 (t0). Prior to NO· addition and at 1-hr post addition, intracellular pH was determined. Additionally, WT *S*. *aureus* was similarly cultured in the absence of NO· in media buffered at decreasing pH. Mid-exponential cultures (OD_650_ = 1.0) were used to determine intracellular pH. **B.** Growth rates of NO·-exposed cultures relative to unexposed in media buffered to the indicated pH. n = 3 for all measurements and significantly reduced growth rates were determined with two-sided Student’s t tests (*, P ≤ .05; **, P ≤ .01).

In order to thrive and sustain many basic cellular processes, cells must maintain proton motive force (PMF), representing the sum of membrane potential (ΔΨ) and pH gradient (ΔpH). PMF is readily generated during bacterial respiration, in which case its energy can be utilized by the F_1_F_0_ ATPase to generate ATP. In contrast, many fermenting bacteria rely on the hydrolysis of ATP by the F_1_F_0_ ATPase coupled to proton extrusion for maintaining PMF [32]. Because *S*. *aureus* lacks identified proton-specific pumps outside its respiratory chain, we predicted that non-respiring *S*. *aureus* might similarly require the F_1_F_0_ ATPase for production of PMF via ATP hydrolysis and proton extrusion. We first assessed intracellular ATP levels (BacTiter Glo) in WT and Δ*atpG S*. *aureus* before and after addition of NO· (Fig 6A). Consistent with fermenting *S*. *aureus* hydrolyzing ATP to extrude protons, the Δ*atpG* mutant exhibited drastically higher levels of ATP compared to WT. This difference was apparent even before the addition of NO·, implying that *S*. *aureus* generally uses the hydrolysis of ATP to defend PMF under respiratory conditions as well (Fig 6A). Next we determined ΔΨ in WT and Δ*atpG S*. *aureus* before and after addition of NO·, reasoning that the inability to extrude protons under fermenting conditions may result in the loss of charge across the membrane. Surprisingly, the Δ*atp*G mutant exhibited a hyperpolarized membrane compared to WT before and after NO· addition (Fig 6B). This result could be explained by *S*. *aureus* compensating for the loss of the proton pumping F_1_F_0_ ATPase by extruding alternative cations other than protons. Defending ΔΨ by extruding alternative cations could result in an inability to specifically export protons efficiently leading to the acidification of the cytosol. Indeed, the Δ*atpG* mutant exhibited significantly reduced intracellular pH compared to WT both before and after NO· addition (Fig 7A).

Interestingly, the intracellular pH of NO·-stressed *S*. *aureus* elevates above 8.0. In contrast, the Δ*atpG* mutant cannot increase intracellular pH above 7.0, even during NO·-stress Consistent with the F_1_F_0_ ATPase operating in the hydrolysis mode, overexpressing the *atpIBEFHAGDC* operon when complementing the Δ*atpG* mutant caused a dramatic increase in intracellular pH that responded to NO· as does WT (Fig 7A). The lower intracellular pH of the Δ*atpG* mutant cannot solely explain the lack of growth in the presence of NO· since the pH does not significantly differ from aerobically cultured mutants, which grow near WT levels (Fig 4A). Rather, we hypothesize that enzymes specifically required for non-respiratory growth are only active at the elevated pHs attained by WT and are essentially non-functional at the mildly acidic intracellular pH of the Δ*atpG* mutant. For instance, the optimal pH for the activity of all three lactate dehydrogenases in *S*. *aureus* is above 8.0 with little measurable activity below 7.5[33]. Accordingly, the Δ*atpG* mutant excretes less lactate per cell than WT when exposed to NO· (S7 Fig). Moreover, while *S*. *aureus* is capable of defending intracellular pH under mild acid stress, a threshold can be reached whereby WT cells are no longer able to maintain optimal cytosolic pH (extracellular pH ≤ 5.5). Below pH 5.5, WT cells exhibit a cytosolic pH similar to that of the Δ*atpG* mutant (Fig 7A). While this reduced intracellular pH does not affect aerobic growth (S6 Fig), it does eliminate the ability of WT *S*. *aureus* to resist NO· (Fig 7B and S6 Fig). These data support our hypothesis that *S*. *aureus* must be able to maintain an alkaline intracellular pH under non-respiratory conditions to allow key metabolic enzymes to operate at maximal efficiency. The Δ*atpG* mutant lacks this ability to alkalinize intracellular pH and therefore cannot replicate efficiently without respiration.

### A functional F_1_F_0_ ATPase is required for *S*. *aureus* persistence and virulence in murine SSTIs

**Fig 8:**
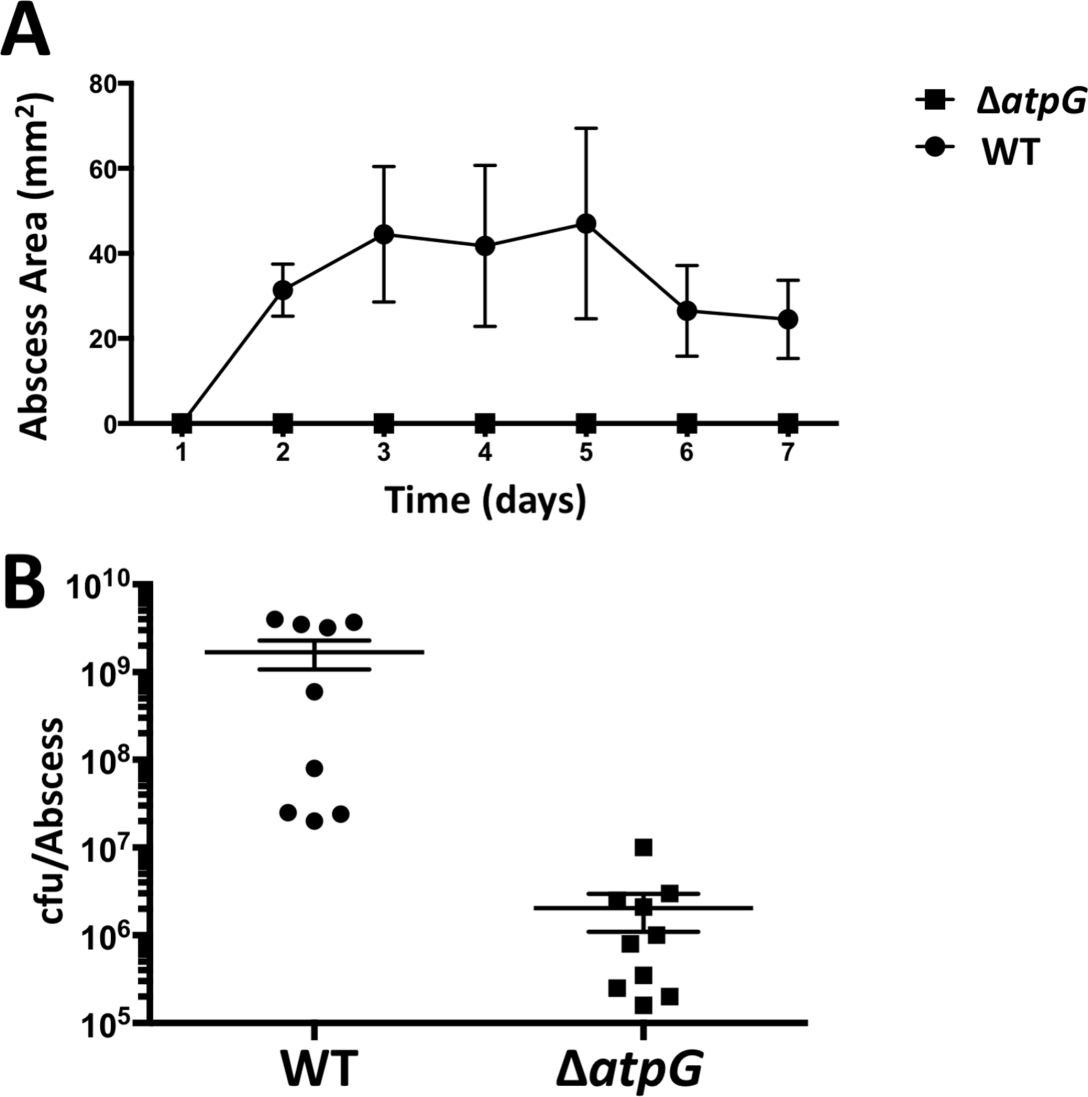
The Δ*atpG* mutant is severely attenuated in a murine skin abscess model. **A.** Abscess area over time; the Δ*atpG* mutant did not cause visible abscesses. **B.** Viable cfu within abscesses 3 days post inoculation.

Given that murine skin abscesses are replete with high NO· levels early on and transition to hypoxia at later time points (both limiting respiration)[7], it is not surprising that we found the F_1_F_0_ ATPase operon to be required for fitness at days 3 and 7 in our Tn-Seq experiment (S3 Table). To verify this result in a non-competitive assay, we infected mice subcutaneously with the Δa*tp*G mutant strain. Indeed, we found the Δ*atpG* mutant to be highly attenuated relative to WT (Fig 8). The mutant forms no visible abscess and is severely reduced in viability 3 days post inoculation. This result underscores the essentiality of the F_1_F_0_ ATPase for CA-MRSA pathogenesis in the skin and reinforces the notion that *S*. *aureus* bacteria persisting in a skin abscess encounter significant respiratory inhibition. Thus, inhibitors targeting the F_1_F_0_ ATPase of *S*. *aureus* are likely to be more potent *in vivo* than they are *in vitro* as the latter is usually tested in aerated cultures[23].

## Discussion

*S*. *aureus* NO· resistance is a complex, multifaceted trait that remains incompletely understood. In the current study, we performed Tn-Seq to broadly screen for genes contributing to fitness during NO· stress. We identified 168 genes specifically required for *in vitro* NO· resistance, many of which had not been previously associated with the NO· response in *S*. *aureus*. We further identified 166 genes specifically required for persistence in murine SSTIs at day 3 and 262 at day 7, of which 49 may be required due to their role in NO· resistance. Of the genes required under both conditions, the most prominent group encode the *S*. *aureus* F_1_F_0_ ATPase, which we show to be essential for the non-respiratory growth of *S*. *aureus*. Additionally, we validated many previous findings by identifying *srrAB*, *qoxABCD*, *ccpA*, *pyk* and *rot* as being essential for full virulence as well as high-level NO· resistance[8,9,12,14]. Interestingly, we did not find mutants with moderate defects in NO· resistance (*e*.*g*., *hmp* and *ldh*1) as having significant defects in murine SSTIs. We have previously observed that many genes contributing to full NO· resistance in *S*. *aureus* have much larger virulence roles in sepsis models than in the SSTI model[34]. While difficult to quantify, it may be that the localized skin immune response generates less NO· than the systemic response to acute sepsis. However, the finding that ~30% of genes required for full NO· resistance *in vitro* are also required for persistence within murine skin infection suggests that immune radicals may still exert some selective pressure in this model. While many of these same gene products confer fitness in other respiration-limited environments as well and may be selected for in the skin for reasons other than NO·, we have shown that hypoxia sets in after day 7 when the wound closes[7]. We did not prolong our Tn-Seq selection beyond day 7 due to the significant loss of bacterial viability that would have imposed population bottlenecks, thereby complicating analyses.

This was the first report to our knowledge of a Tn-Seq screen performed with a CA-MRSA isolate of *S*. *aureus*. We observed many differences in the fitness requirements in SSTIs using *S*. *aureus* LAC compared to a recent study that used the strain HG003, a MSSA strain[20]. For example, the authors of this study found that a primary group of genes required for fitness at 48-hrs in SSTIs but not rich media were pyrimidine biosynthetic genes. In contrast, we did not observe any differences in fitness for the majority of pyrimidine biosynthetic genes between rich media and SSTIs. Furthermore, the former study found *sarA* Tn-insertion mutants to be supergrowers in SSTIs. This is in stark contrast to our study, in which *sarA* Tn-insertion mutants were highly compromised for fitness both *in vitro* and *in vivo*. Many other studies also support a role for *sarA* in *S*. *aureus* virulence and fitness, corroborating our result[35,36]. Finally, the previous report found a modest defect in fitness within murine abscesses for mutants in *agrA*, whereas we found insertional inactivation of the Agr system to improve fitness. This implies that in LAC, a strain known to robustly express Agr, production of numerous virulence factors imparts a fitness cost that can be overcome in mixed infections when the bulk bacterial population is Agr-positive. This could also explain the reduced fitness of insertions in *codY*. While Δ*codY* mutants have been reported to be hypervirulent[37], presumably due to heightened Agr-activity[38], in a mixed infection enhanced Agr-activity could impose a fitness cost.

These discrepancies are likely due to major differences in virulence between LAC and HG003. LAC proliferates much more extensively in murine SSTIs than HG003. Moreover, relative to other clinical isolates, CA-MRSA strains have been shown to produce elevated levels of alpha-toxin, PSMs, and secreted proteases[4], all of which would increase inflammation and host cell lysis while decreasing the ability of the host to confine the lesion. As a result, the skin abscess environments encountered by LAC and HG003 would be very different. Differences in host cell lysis by *S*. *aureus* toxins would alter the pool of available nutrients and thus differentially affect the metabolic genes required for fitness. Furthermore, disparities in abscess morphology and inflammation would have major impacts on oxygen availability and levels of antimicrobial inflammatory mediators, also influencing which genes contribute to fitness for each strain. Additionally, CA-MRSA strains have altered virulence gene regulation compared to many HA-MRSA strains. Therefore, elevated expression of certain genes could greatly increase their relative importance during infection. The dissimilarities in results between the two Tn-Seq studies emphasize the importance of recognizing strain differences in *S*. *aureus* research and not generalizing results between strains.

**Fig 9:**
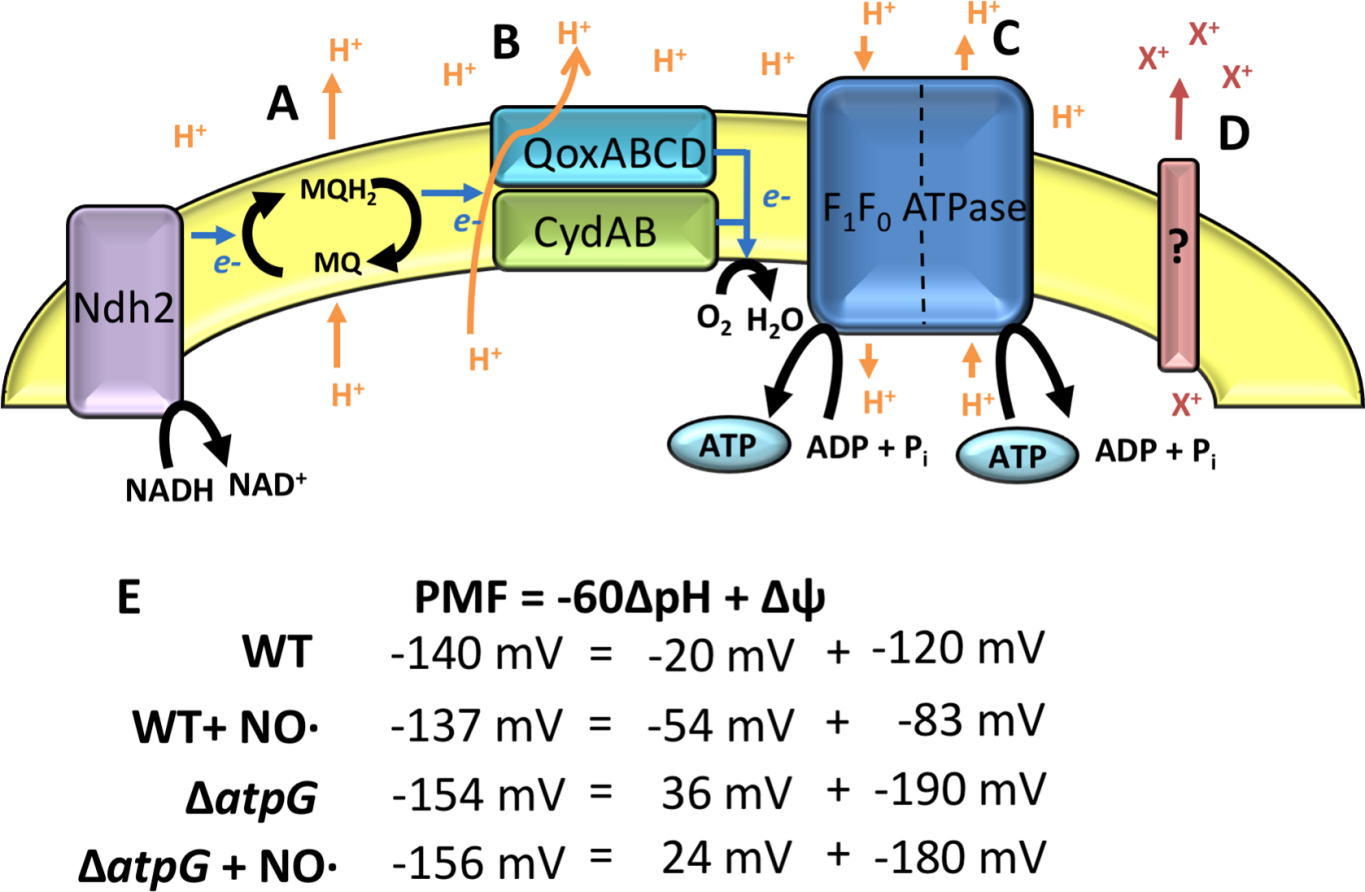
Both WT and Δ*atpG* are capable of defending their PMF in the presence of NO·. Schematic of the respiratory chain of *S*. *aureus* depicts elements that contribute to PMF as **A**. Q-loops, **B**. active pumping by cytochrome *aa*_3_, **C**. reversal of the F_1_F_0_ ATPase, or **D.** transporters of other non-proton cations (which would contribute to Δψ but not ΔpH). **E.** Calculation of the different components of PMF in WT and Δ*atpG* in the presence/absence of NO·.

The most significant finding was the absolute necessity of the F_1_F_0_ ATPase for *S*. *aureus* growth in the absence of respiration and during infection. This was fully consistent with previous Tn-Seq approach reports [20]. Our initial hypothesis to explain this finding was that this enzyme complex was the only means of establishing PMF in non-respiring cells. PMF is an important cellular energy source used to perform work such as ATP synthesis and solute transport, and it is commonly generated during respiration at coupling sites in the electron transport chain where protons are pumped out of the cell. The only predicted proton pump in the *S*. *aureus* respiratory chain is the terminal oxidase QoxABCD (Fig 9B). *S*. *aureus* possesses a type II NADH dehydrogenase that does not translocate protons[39]. Likewise, the high affinity terminal oxidase CydAB and nitrate reductase NarGH are also predicted to lack proton translocation activity. However, respiring cells can accomplish proton translocation via Q loops, when quinones are reduced on the cytoplasmic side of the cell membrane and acquire protons that are subsequently released extracellularly when quinol oxidation occurs on the opposite side of the membrane(Fig 9A)[40–42]. In the absence of respiration, proton extrusion via Qox or Q loops does not occur and PMF must be maintained in other ways. In many bacteria including *E*. *coli*, a major strategy for translocating protons in the absence of respiration is reversal of the F_1_F_0_ ATPase reaction, where ATP is hydrolyzed for energy to translocate protons out of the cell[32]. We therefore proposed that the F_1_F_0_ ATPase is necessary for functioning as a proton pump in the absence of respiration to contribute to PMF homeostasis (Fig 9C). Consistent with this notion is the fact that ATP levels in the Δ*atpG* mutant were always higher than WT suggesting that the F_1_F_0_ ATPase is a major consumer of ATP rather than a source. However, the Δ*atpG* mutant was able to maintain PMF and even exhibited a hyperpolarized membrane suggesting that a compensatory ion (*e*.*g*. K^+^ or Na^+^) was being used to maintain ΔΨ (Fig 9D)[43]. In this case, the compensatory ion would only contribute to ΔΨ and not ΔpH. Therefore, to attain constant PMF, a hyperpolarized membrane (higher ΔΨ) would be necessary. This would explain the enhanced susceptibility of the Δ*atpG* mutant to aminoglycoside antibiotics (*e*.*g*. kanamycin, Fig 5C) given that these drugs rely on ΔΨ to drive uptake[44]. Another consequence of exporting cations other than protons is that the cytosol would become acidic since protons are not efficiently extruded. Indeed, WT exhibited raised pH upon exposure to high NO· levels sufficient to inhibit respiration, whereas the cytosol of the Δ*atpG* mutant remained slightly acidic after NO· addition. This is problematic for enzymes that are critical for maintaining redox balance when respiration is inhibited, namely the three lactate dehydrogenases of *S*. *aureus*, which exhibit maximal activity above pH 8.0[33]. The acidic cytosol of the Δ*atpG* mutant would limit the activity of these enzymes, and potentially others that are critical to non-respiratory growth. The result is that the Δ*atpG* mutant simply cannot thrive under conditions in which respiration is limited.

In other bacterial species, the importance of the F_1_F_0_ ATPase varies and has been attributed to a variety of mechanisms including meeting energy demands, balancing pH and serving as a proton “relief valve” [45–47]. Thus, the requirement for the F_1_F_0_ ATPase in non-respiring bacteria is not specific to *S*. *aureus* as it was also shown to be essential for fermenting *Listeria monocytogenes*[22]. Regardless of the role for the F_1_F_0_ ATPase in individual bacteria, targeting it with novel antimicrobials may represent a broad-spectrum treatment option. Importantly, there have been recent attempts to develop compounds that specifically target bacterial F_1_F_0_ ATPases, including diarylquinolone drugs specific for the *Mycobaterium tuberculosis* F_1_F_0_ ATPase that have been entered into phase IIb clinical trials[48,49]. While these compounds are fairly specific for mycobacteria, other derivatives developed to target Gram positive F_1_F_0_ ATPases have recently been reported as having bactericidal activity on both planktonic and biofilm-associated *S*. *aureus*[23]. Here, our results suggest that targeting the F_1_F_0_ ATPase may prove to be very effective *in vivo* given the environmental factors that limit bacterial respiration at sites of inflammation (*e*.*g*. iron limitation, hypoxia and immune radicals).

By applying a non-biased saturating Tn-Seq approach to identify *S*. *aureus* NO·-resistance genes that are critical for fitness within a mammalian SSTI, we have both validated previous reports describing enzymes and regulators essential for full NO· resistance in *S*. *aureus* and at the same time identified new genes. Future work will focus on illuminating the mechanisms by which these new genes contribute to NO· resistance in *S*. *aureus* as well as enhance fitness within the inflamed skin abscess environment.

## Materials and methods

### Ethics statement

Animal studies carried out in this work fall under an animal protocol approved by the University of Pittsburgh Institutional Animal Care and Use Committee (protocol id 16027663, PHS Assurance number: A3187-01). The University of Pittsburgh is an AAALAC accredited institution and adheres to the standards set by the Animal Welfare Act and the NIH Guide for the Care and Use of Laboratory Animals.

### Strains and growth conditions

Bacterial strains, plasmids, and primers are listed in S4 Table. *S*. *aureus* LAC, a USA300 isolate, was used as background for all experiments. LAC Tn-library construction is described below. Deletion mutants to verify Tn-Seq results were created via allelic exchange using previously described methods[50,51]. TSB containing 5mg/ml glucose (achieved by supplementation with an additional 2.5mg/ml glucose) was used for *in vitro* growth experiments to ensure glucose was not limiting given the its requirement for NO· resistance[9].

Growth curves were performed using 200μl cultures within a 96-well plate. A Tecan Infinite M200 Pro microplate reader was used to detect change in absorbance (650nm) at 15-min intervals. Growth curves were run for 24-hrs, or 96 cycles of 1mm orbital shaking for 830s followed by 1mm linear shaking for 30s. Overnight cultures were grown in TSB 5mg/ml glucose, washed with PBS, and diluted to an OD_650_ of 0.01 for each growth curve. The NO· donors used in this study were 2,2′-(hydroxynitrosohydrazono)bis-ethanimine (DETA)/NO, or a mixture of NOC-12 and diethylamine nitric oxide (DEA/NO), each resuspended in 0.01 N NaOH.

### Transposon library construction

The transposon library was also constructed using *S*. *aureus* LAC. For generation of the transposon library, we used a modified version of the plasmids and protocol for *bursa aurealis* transposition in *S*. *aureus* described previously[25]. We modified the plasmid pFA545 by replacing the xylose-inducible promoter and transposase allele (*tnp*) (contained within the *Nhe*I-digestible fragment) with a new fragment containing the constitutive *lgt* promoter fused to *tnp* to form pMG020. This plasmid was transformed into RN4220 and a Φ-11 phage lysate was generated immediately for future steps. A *S*. *aureus* LAC strain containing pBursa was transduced with pMG020 Φ-11 phage lysate, incubated at 30° C, and individual colonies from the transduction were resuspended in 100μl PBS. 10–15μl of this resuspension was plated on large petri dishes (150mm) of TSB containing 10μg/ml erythromycin and incubated at 43° C for 48-hrs to allow for transposition to occur. 60 plates of approximately 2,500 colonies were each scraped by adding 2ml of TSB 10μg/ml erythromycin + 25% glycerol. Each aliquot was thoroughly vortexed and then 1ml from each was combined into a single pool representing approximately 150,000 transposon mutants (~2 colonies for each individual Tn insertion). 100μl aliquots of this pool were frozen at -80° C until use. Before using any aliquots for experiments, one aliquot was thawed, and DNA was extracted using the Epicentre MasterPure Gram Positive DNA Purification Kit and subjected to Tn-Seq Analysis.

### Selection experiments

To verify an assay in which NO·-sensitive mutants could be selected against *in vitro*, we mixed known NO·-sensitive mutants (Δ*srrAB*, Δ*hmp*, Δ*ldh1*, and Δ*sarA*) of varying sensitivities with WT LAC at a ratio of 1:100 (more representative of a Tn-Library in which most of the culture will not be NO· sensitive). Cultures were diluted to a starting OD_650_ 0.01 (10^7^ cfu/ml) containing a 1:100 ratio of mutant to WT in 5ml of TSB 5mg/ml glucose. For NO· selection, 10mM DETA/NO was added at inoculum, and every 12-hrs for a 48-hr period cultures were diluted 1:100 into fresh, warm TSB 5g/L glucose plus 10mM DETA/NO. For aerobic selection, cultures were grown in the absence of DETA/NO and were diluted 1:100 every 5-hrs for a 15-hr period. At each time point, serial dilutions of the cultures were plated both on plain TSB and TSB containing appropriate antibiotics to select for each mutant, allowing for the ratio of mutant:WT to be plotted over time.

### Tn-library selection experiments

For all experiments using the Tn-library, a 100μl aliquot of library was thawed and then added to 100ml of TSB 5g/L glucose and grown for short overnight of 10-hrs (to minimize selection during stationary phase). Composition of this inoculum culture was also analyzed by Illumina sequencing. For selection in the presence of NO·, an overnight culture was diluted to an OD_650_ 0.02 (approximately 10^7^ cfu/ml) in 5ml of TSB 5mg/ml glucose, and 5mM DETA/NO was added. The culture was grown shaking at 37° C. Every 12-hrs for a 48-hr period, the cultures were diluted 1:100 into fresh, warm TSB 5g/L glucose and fresh 10mM DETA/NO was added. Additionally, at each 12-hr dilution time point, the culture was plated for cfu to determine the number of generations that had occurred; on average there were 6–7 generations per 12-hrs for a total of 24–28 generations per experiment. For selection during aerobic growth, a starting OD_650_ 0.02 was used and cultures were grown shaking at 37° C but in the absence of DETA/NO. To achieve approximately the same number of generations per serial passage as during growth in the presence of DETA/NO, cultures were diluted 1:100 into fresh TSB every 5-hrs for a 20-hr period and again plated for cfu at each 5-hr time point.

For selection in murine SSTIs, six-week old C57/B6 mice were infected as previously described[34]. Briefly, mice were shaved and anesthetized with avertin (250 mg/kg), and inoculated subcutaneously with 10^7^ cfu (20μl of overnight diluted in PBS to an OD_650_ 1). Four mice were sacrificed each at day 3 and day 7 post-infection. To collect bacteria from abscesses, we modified a recently published procedure[20]. Abscesses were removed and pushed through mesh 40 μm nylon cell strainers (Falcon)for homogenization, using 1.5ml TSB + erythromycin. Each 1.5ml tissue homogenate was diluted 1:10 into 15ml of TSB erythromycin and a 5-hr outgrowth was allowed by shaking at 37° C. After outgrowth, the cultures were centrifuged to pellet bacteria and samples were pooled into two groups (each representing two mice) to ensure enough bacteria for DNA extraction. Each pellet was resuspended in 1ml TSB Erythromycin + 25% glycerol and frozen at -80° until DNA extraction.

### Library prep for Tn-Seq

DNA was extracted from each library using the Epicentre MasterPure Gram Positive DNA Purification Kit (Madison, WI). DNA was then fragmented by nebulization to a range of ~300-700bp fragments using Rapid Nebulizers (454 Life Sciences, Branford, CT) according to manufacturer instructions, followed by purification using a QIAquick PCR Purification Kit (Qiagen). Libraries were prepped for sequencing according to a previously published protocol with minor modifications[52]. Poly-C tails were added to 1μg of fragmented DNA using terminal deoxynucleotidyl transferase (Promega, Madison, WI) in a 20μl reaction (47.5μM dCTP, 2.5μM ddCTP, 0.5μl TdT enzyme, 4μl 5x buffer) run for 1-hr at 37° C. After the reaction, products were purified using Edge Biosystems spin columns (Gaithersburg, MD). An initial PCR was performed to specifically amplify transposon-genome junctions with a poly-G primer (olj376) and a transposon specific primer (olj510). The 50μl PCR reaction contained 5μl poly-C DNA, 1.8μM primer olj376, 0.6μM primer olj510, 0.4mM dNTPs, 1μM Easy-A Cloning Enzyme, and 5μl 10x buffer. The thermocycler was programmed as follows: cycle 1 (1x, 1-min at 95° C), cycle 2 (25x, 30-sec at 95° C, 30-sec at 58° C, 2-min at 72° C), and cycle 3 (1x, 2-min at 72° C). For additional specificity and to add Illumina adapter sequences and barcodes, a second PCR was performed with a nested transposon-specific primer (olj511) and standard Illumina barcoding primers. The 50μl PCR reaction contained 0.5μl from first PCR reaction, 0.6μM primer olj511, 0.6μM barcode primer, 0.4mM dNTPs, 1μM Easy-A Cloning Enzyme, and 5μl 10x buffer. The thermocycler was programmed as follows: cycle 1 (1x, 1-min at 95° C), cycle 2 (15x, 30-sec at 95° C, 30-sec at 52° C, 2-min at 72° C), and cycle 3 (1x, 2-min at 72° C). To remove primer-dimers and further size-select samples, we used Agencourt AMPure XP beads to purify PCR products. Prepped libraries were then multiplexed and sent for sequencing on an Illumina HiSeq2500 using a custom primer (olj512) and 50x unpaired reads.

### Tn-Seq analysis

Reads were mapped to the *S*. *aureus* USA300_FPR3757 genome with Geneious 8.0 using medium sensitivity over three iterations to generate.sam files for further analyses. The program Tn-Seq Explorer[53] was used for tabulating read counts per Tn-insertion, insertion number and insertion density (# of actual insertions/# of possible insertions) for each gene. Insertion density takes into account both gene length and AT content, both of which affect the number of insertions possible. Median read per insertion was determined for each gene using a python-based Tn-Seq code (available upon request). There were a significant number of overrepresented reads in the output libraries from murine SSTIs. To exclude these “jackpots” from the analysis, we used median read counts per gene rather than total or average read counts. We generated “representation” or “R” values for each gene, where R = (median read count)x(insertion density), thereby eliminating any influence from random jackpots. To examine the relative importance of every gene for fitness within each library, log transformed R values for each data set were used to define essential genes ≤ 3 SD below the mean R-value or genes that significantly contribute to fitness (3 SD ≤ R-value ≤ 2 SD below mean).

Ratios between R-values of NO· cultured cells to aerobically cultured cells were calculated. First, all genes deemed essential for serial aerobic passage (≤ 3 S.D. below median log-transformed R-value) were eliminated. The remaining were used to compare R-values between NO· and aerobic cultures. Similarly, ratios between day 3 and day 7 compared to overnight cultures were generated by initially removing genes essential for overnight growth.

### Complementation of Δ*atpG*

Since *atpG* is located roughly in the middle of the *atpIBEFHAGDC* operon and thus is likely polar on the distal genes, we complemented the Δ*atpG* operon with pEP01 harboring the entire operon under its native promoter on pLZ-Spec. A 7.2 kb fragment containing the *atpIBEFHAGDC* operon and its promoter were amplified with atp.1a/1b (S4 Table) and cloned into the *Nde*I/*Xho*I sites in pLZ-Spec. The resulting pEP01 was electroporated into AR1524 (LAC Δ*atpG*) to yield the complemented strain, AR1591.

### Growth curves

Aerobic, NO·, and iron chelation growth curves were performed as described above in 96-well plates on a Tecan M200 plate reader. For NO· exposure, strains were treated with 10mM DETA/NO at the time of inoculation or with the combination of 10mM NOC-12/ 1mM DEA/NO at mid-exponential phase (OD_650_ = ~0.2). For iron chelation, strains were treated with 2mM 2,2’-dipyridyl at the time of inoculation. Anaerobic growth curves were performed using a BioTek Synergy H1 plate reader with oxygen control. The percent oxygen was maintained at 1% throughout the experiment with the dissolved oxygen being even lower.

### MICs

Overnight cultures of WT and Δ*atp*G grown in TSB 5mg/ml glucose were diluted to an OD_650_ 0.01 in 96-well plates. Two-fold serial dilutions of H_2_O_2_, kanamycin, vancomycin, tetracycline, and chloramphenicol were added to wells of each strain at the time of inoculation. Cultures were shaken at 37° C in a Tecan M200 plate reader for 18-hrs. Minimum inhibitory concentration (MIC) was defined as the minimum amount of reagent required to inhibit growth above OD_650_ 0.15 for 18-hrs.

### ATP quantification

Intracellular ATP concentrations were measured for WT and Δ*atp*G using the BacTiter-Glo microbial cell viability assay (Promega) according to manufacturer instructions. ATP levels were normalized to the OD_650_ value for each time point. Strains were grown in TSB 5mg/ml glucose in 96-well plates, and aerobic ATP levels were measured at an OD_650_ 0.25, at which time NO· mix (10mM NOC-12, 1mM DEA/NO) was added to remaining wells (t0). ATP levels were again measured 1-hr following NO· addition.

### Membrane potential and intracellular pH measurements

Membrane potential was measured for WT and Δ*atp*G using the BacLight Bacterial Membrane Potential Kit (ThermoFisher). Strains were grown in TSB 5mg/ml glucose in 96-well plates and membrane potential was measured at OD_650_ 0.25 (t0), just before NO· addition, and then 1-hr following NO· mix (10mM NOC12, 1mM DEANO) addition. At each time point, two 200μl wells of each strain were combined and concentrated in half the volume of PBS (200μl) and transferred to a 96-well black, clear-bottom plate. 30μM DiOC_2_(3) dye was added to the concentrated culture. Using a Tecan M200 plate reader, red and green fluorescence was detected (emission 488, excitation 525 and 613) every 5-min for a 30-min period. The maximum red:green fluorescence ratio was taken as the value for relative ΔΨ. Fluorescence ratios were converted to mV by interpolating data from a standard curve generated by addition of 1 μM valinomycin plus K^+^ at the following concentrations (μM): 0, 1, 3, 10, 30, 100 and 300. These correspond to a ΔΨ of (mV): -180, -150, -120, -90, -60, -30 and 0, respectively.

Intracellular pH was determined for WT, Δ*atpG* and Δ*atpG* + pATPase using the pHrodo Red AM Intracellular pH Indicator Kit (ThermoFisher). Strains were grown in TSB 5g/L glucose to an OD_650_ 0.25 then exposed to NO· mix (10mM NOC12, 1mM DEANO) for 1-hr. At 1-hr post. 200μl of each sample was collected and washed with HEPES buffer pH 7.4 and then stained with 50 nM pHrodo Red AM staining solution and incubated at room temperature for 30 minutes. Samples were then washed with HEPES buffer pH 7.4 and fluorescence measurements were taken using a BioTek Synergy HI plate reader (excitation 550, emission 590). Fluorescence measurements were converted to pH values using a standard curve of samples treated with 10μM valinomycin/nigericin at pH levels 4.5, 5.5, 6.5, and 7.5.

## Supporting information

S1 Fig Constitutive transposase expression does not result in multiple insertion sites per genome. Southern blot of *Cla*I digested chromosomal DNA from 16 randomly chosen transposon mutants probed for the presence of transposon sequence.
(TIF)

S2 Fig Density of transposon insertions within the *S*. *aureus* genome. Median distance between transposon insertions is not subject to elevated estimates of means due to long regions of essential DNA or reduced estimates from the 1066 insertions at the identical site on opposing strands (0 bp between insertions).
(TIF)

S3 Fig Venn diagram of genes essential to *S*. *aureus* reported by three independent studies. Comparing our results with those from Valentino, MD *et al*. 2014 *mBio* and Chaudhuri, RR *et al*. 2009 *BMC Genomics* reveals significant overlap. Many of the genes found only in one study are genes specific to the strain used for mutagenesis (HG003 in Valentino *et al*., SH1000 in Chaudhuri *et al*. and LAC in this study.).
(TIF)

S4 Fig Technical and biological replicates for Tn-Seq are highly reproducible. Transposon insertion densities for each gene, which are calculated as # of actual insertion sites per # of possible insertion sites, are plotted for each technical and biological replicate and indicate a high degree of reproducibility.
(TIF)

S5 Fig Genes that encoded subunits of the F_1_F_0_ ATPase are drastically underrepresented from NO·-exposed cultures. Insertion location and read coverage (height of bars) in one replicate of our Input pool (overnight culture), Aerobic culture (24 generations in shaking culture) or NO· culture (24 generations in the presence of NO·-donor).
(TIF)

S6 Fig Representative growth curves from WT *S*. *aureus* with/without NO· exposure cultured at indicated buffered pH. **A.** Growth of WT *S*. *aureus* LAC aerobically in TSB buffered to indicated pH. **B.** Growth of WT *S*. *aureus* LAC in TSB buffered to the indicated pH in the presence of NO· (10 mM NOC-12/1mM DEA-NO added at indicated time). As extracellular pH is dropped, the concomitant drop in intracellular pH inhibits growth specifically during NO· stress.
(TIF)

S7 Fig Defective lactate excretion from ∆*atpG* after NO·-exposure. Both L- and D-lactate levels were determined before and 2-hr after NO· addition (10 mM DETA/NO) for both the WT and ∆*atpG* mutant and normalized to the change in OD_650_ over that same time period. The mutant consistently excreted 50% of the L-lactate and 33% of the D-lactate normally secreted by WT. Statistical significance was determined using Student’s t-test (n = 3, * p ≤ 0.05, ** p ≤ 0.01).
(TIF)

S1 Table Comparison of current Tn-Seq data with previous studies. Genes deemed essential (Red) in the current study as well as those that significantly contribute to fitness (Orange) are listed side by side with results from Valentino MD *et al*. and Chaurdhuri RR *et al*.
(XLSX)

S2 Table List of genes and R-values for each test condition. Each gene is listed in genomic order with the averaged R-values (between duplicates) for Input_Library (colonies from original Tn plate), Overnight (10-hr overnight cultures), Aerobic (24 generations in shaking culture tubes), NO· (24 generations in shaking culture tubes supplemented with 5 mM DETA/NO) as well as day 3 and day 7 SSTI R-values.
(XLSX)

S3 Table R-value ratios comparing NO·-cultured libraries to aerobically cultured libraries. Genes are sorted from lowest to highest NO·:Aerobic R-value ratios. Orange cells represent genes with log-transformed R-values ≤ 3 SD below mean, Green cells represent genes with R-value ratios between 2 and 3 SD below the mean, Dark-grey cells represent genes with R-value ratios between 2 and 3 SD above mean and Light-grey cells represent genes with R-values ≥ 3 SD above the mean.
(XLSX)

S4 Table R-value ratios comparing day 3 and day 7 murine SSTI libraries to overnight culture libraries. Day 3 and Day 7 data are listed in separate tabs. Genes are sorted from lowest to highest SSTI:overnight R-value ratios. Orange cells represent genes with log-transformed R-values ≤ 3 SD below mean, Green cells represent genes with R-value ratios between 2 and 3 SD below the mean, Dark-grey cells represent genes with R-value ratios between 2 and 3 SD above mean and Light-grey cells represent genes with R-values ≥ 3 SD above the mean.
(XLSX)

S5 Table Primers, plasmids and bacterial strains used in this study. (XLSX)
