# Supplementary figures and images for: Genetic requirements for Staphylococcus aureus nitric oxide resistance and virulence
Source: PLoS Pathog. 2018 Mar 19;14(3):e1006907. doi: 10.1371/journal.ppat.1006907 (PMC5884563; doi:10.1371/journal.ppat.1006907)

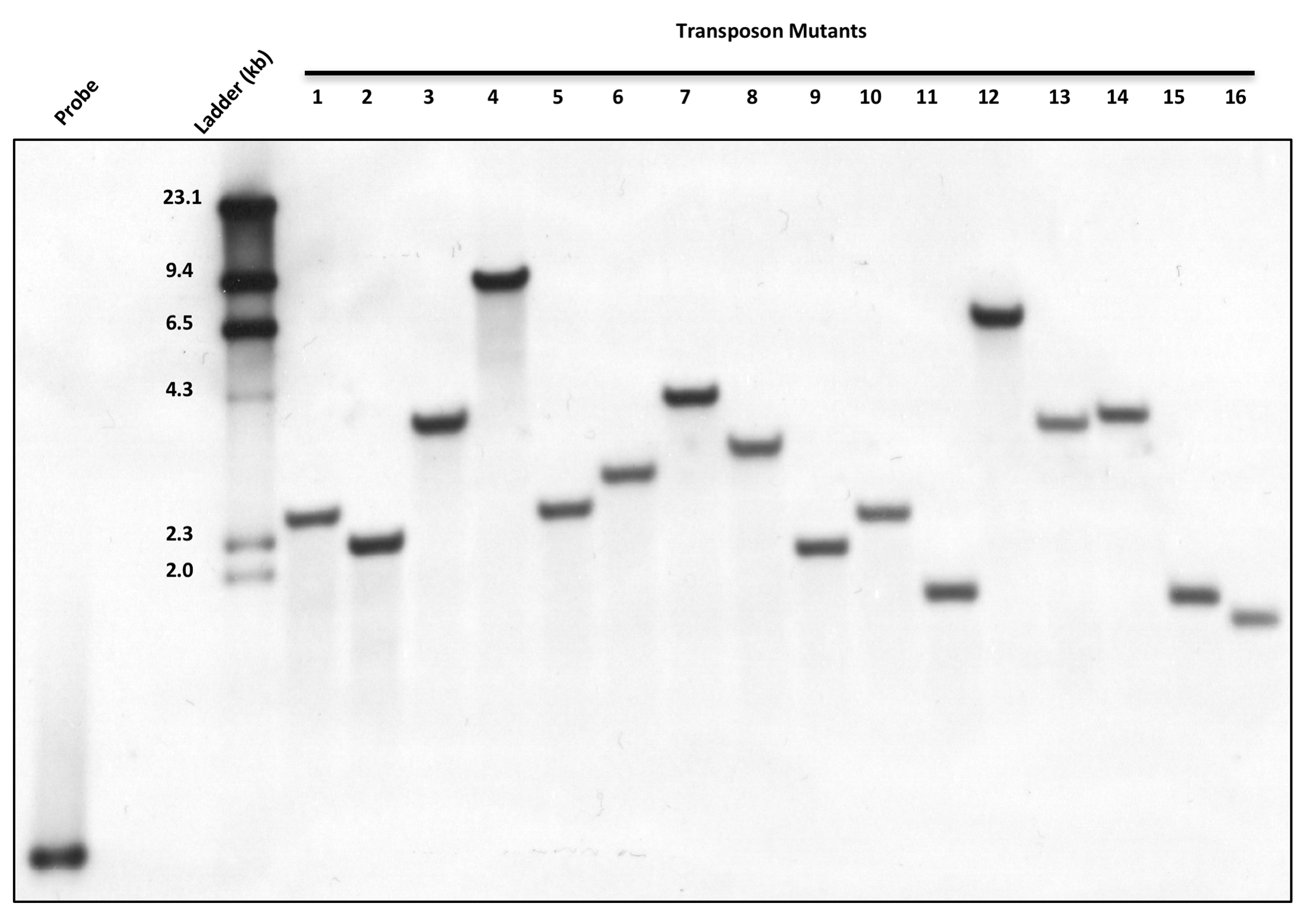

Supplement: S1 Fig — Southern blot of ClaI digested chromosomal DNA from 16 randomly chosen transposon mutants probed for the presence of transposon sequence. (TIF) [file ppat.1006907.s001.tif]

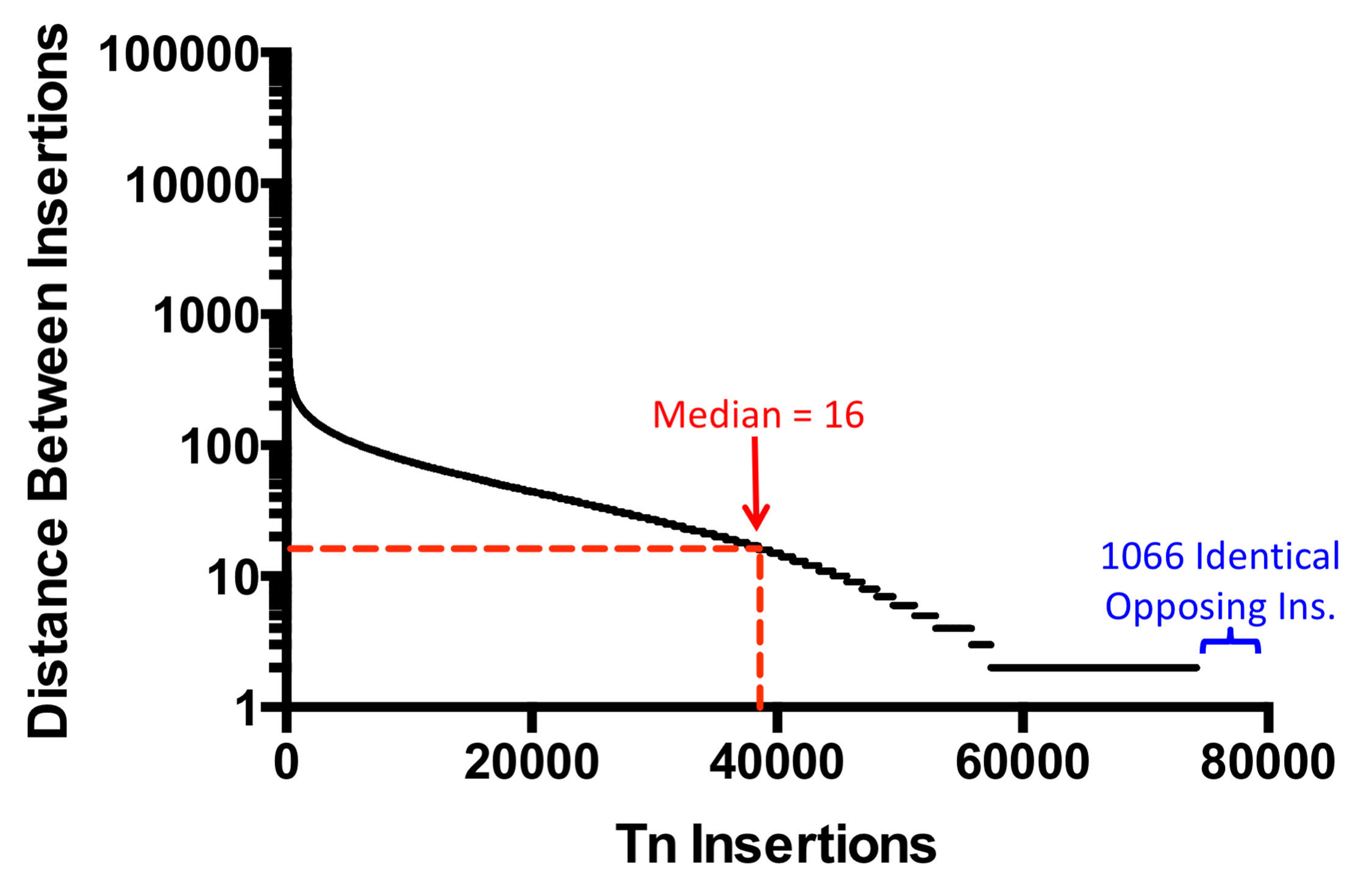

Supplement: S2 Fig — Median distance between transposon insertions is not subject to elevated estimates of means due to long regions of essential DNA or reduced estimates from the 1066 insertions at the identical site on opposing strands (0 bp between insertions). (TIF) [file ppat.1006907.s002.tif]

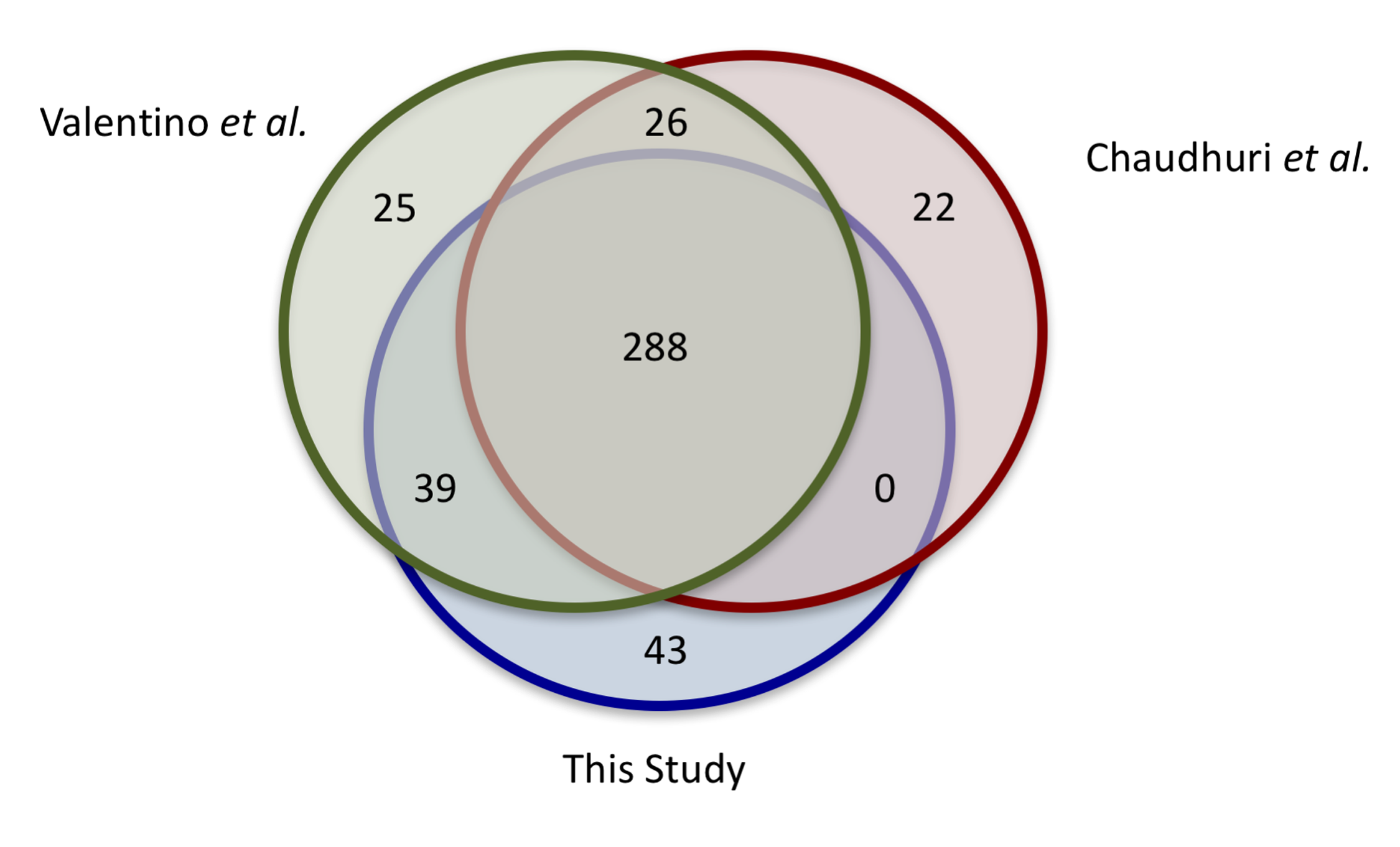

Supplement: S3 Fig — Comparing our results with those from Valentino, MD et al. 2014 mBio and Chaudhuri, RR et al. 2009 BMC Genomics reveals significant overlap. Many of the genes found only in one study are genes specific to the strain used for mutagenesis (HG003 in Valentino et al., SH1000 in Chaudhuri et al. and LAC in this study.). (TIF) [file ppat.1006907.s003.tif]

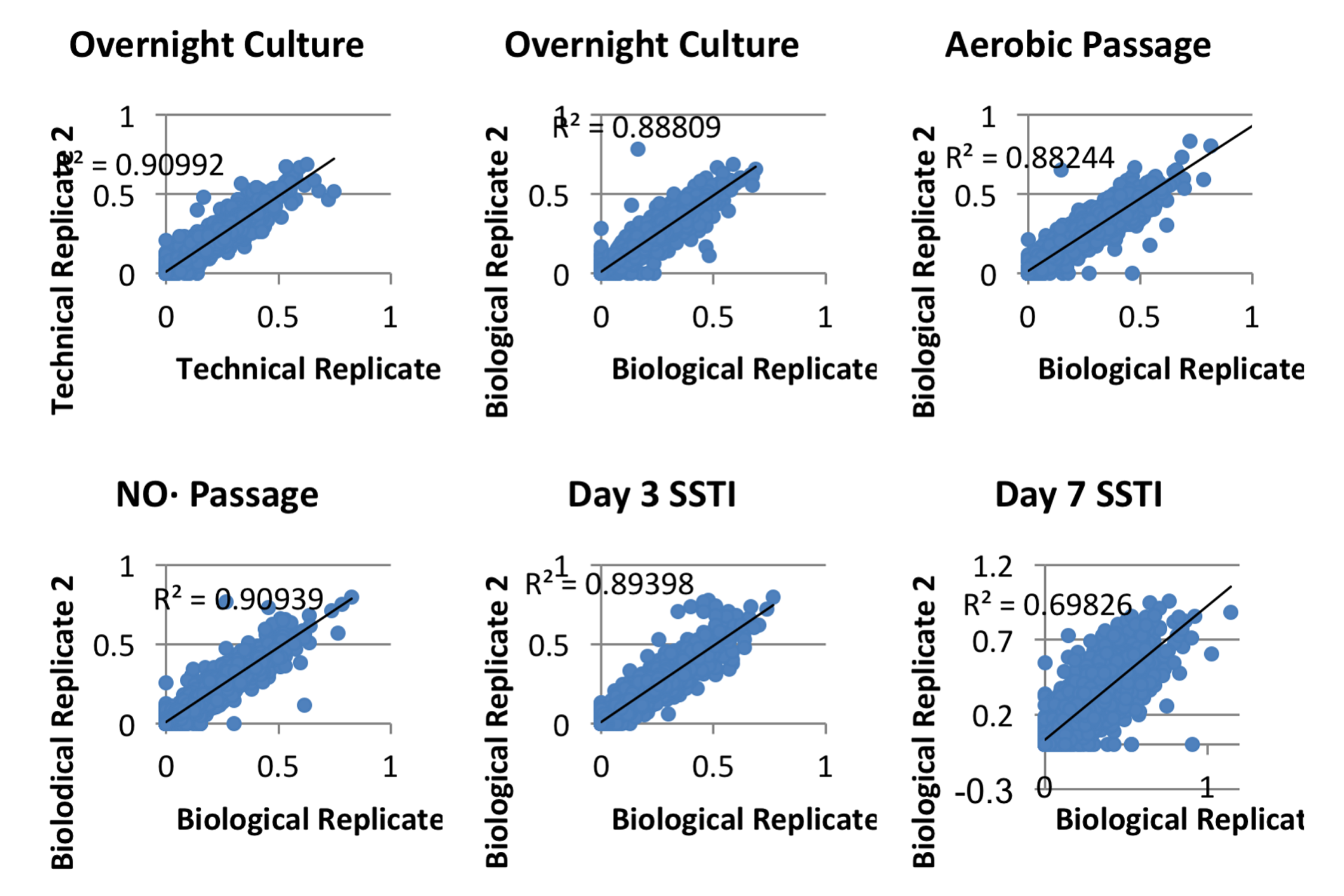

Supplement: S4 Fig — Transposon insertion densities for each gene, which are calculated as # of actual insertion sites per # of possible insertion sites, are plotted for each technical and biological replicate and indicate a high degree of reproducibility. (TIF) [file ppat.1006907.s004.tif]

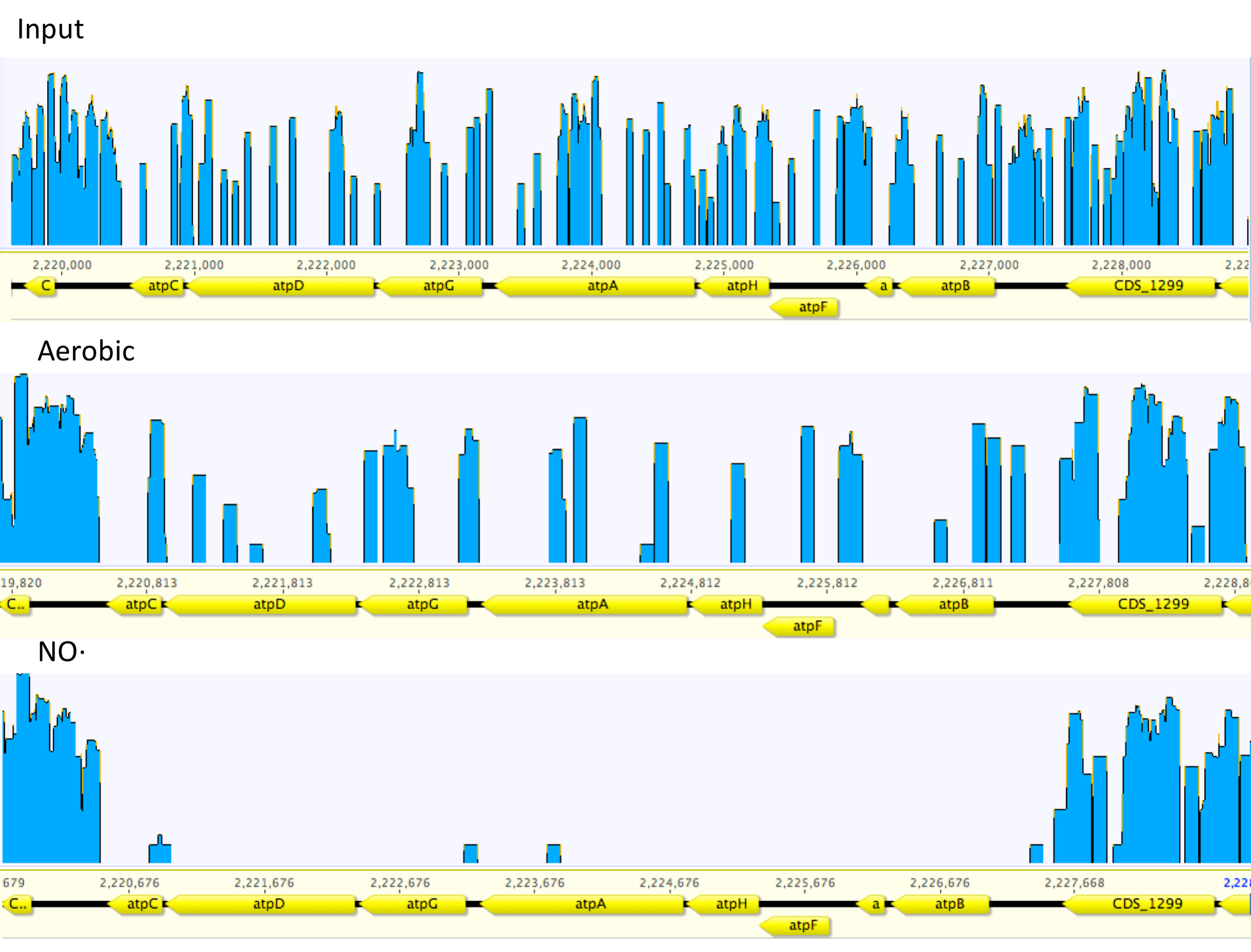

Supplement: S5 Fig — Insertion location and read coverage (height of bars) in one replicate of our Input pool (overnight culture), Aerobic culture (24 generations in shaking culture) or NO· culture (24 generations in the presence of NO·-donor). (TIF) [file ppat.1006907.s005.tif]

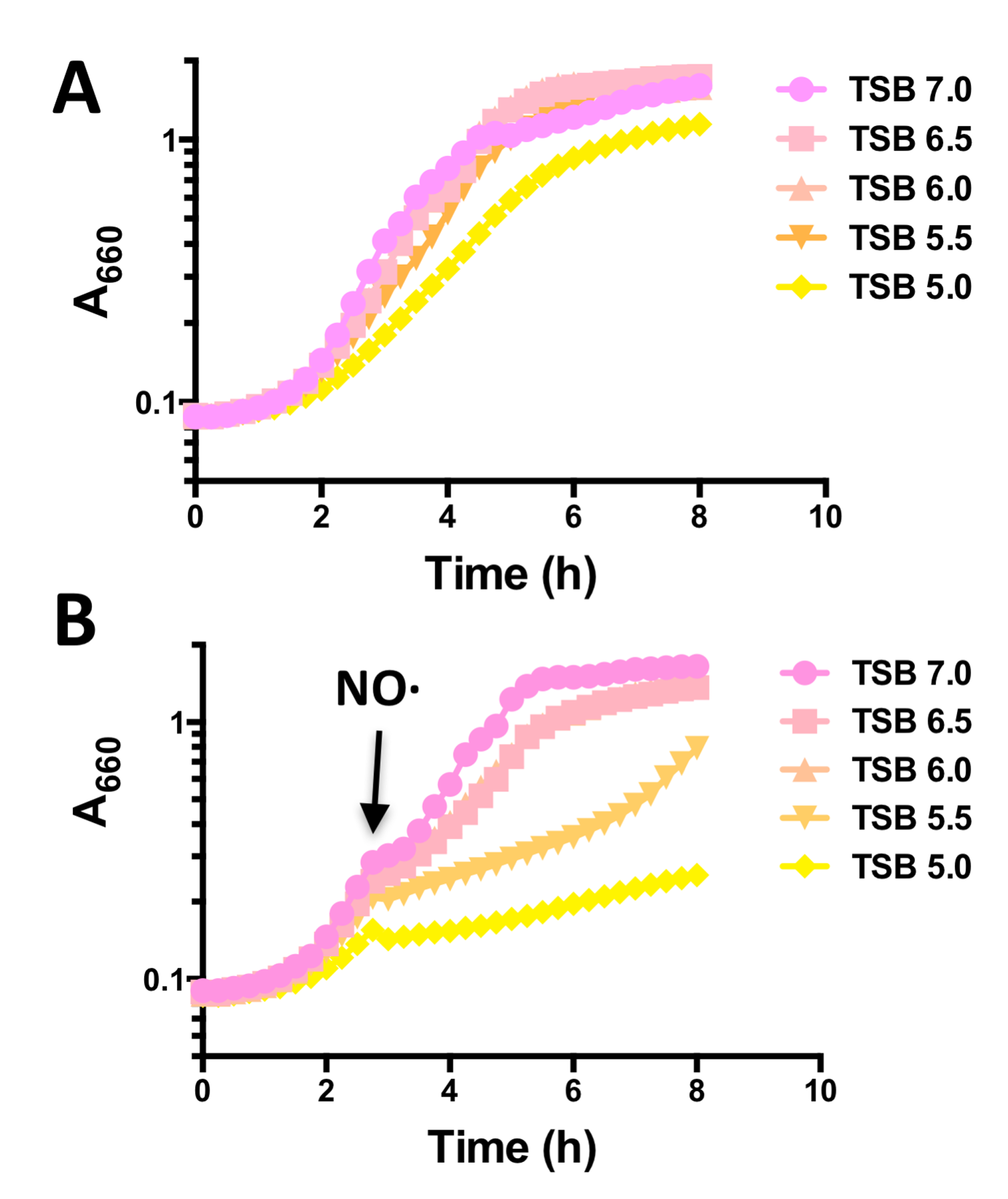

Supplement: S6 Fig — A. Growth of WT S. aureus LAC aerobically in TSB buffered to indicated pH. B. Growth of WT S. aureus LAC in TSB buffered to the indicated pH in the presence of NO· (10 mM NOC-12/1mM DEA-NO added at indicated time). As extracellular pH is dropped, the concomitant drop in intracellular pH inhibits growth specifically during NO· stress. (TIF) [file ppat.1006907.s006.tif]

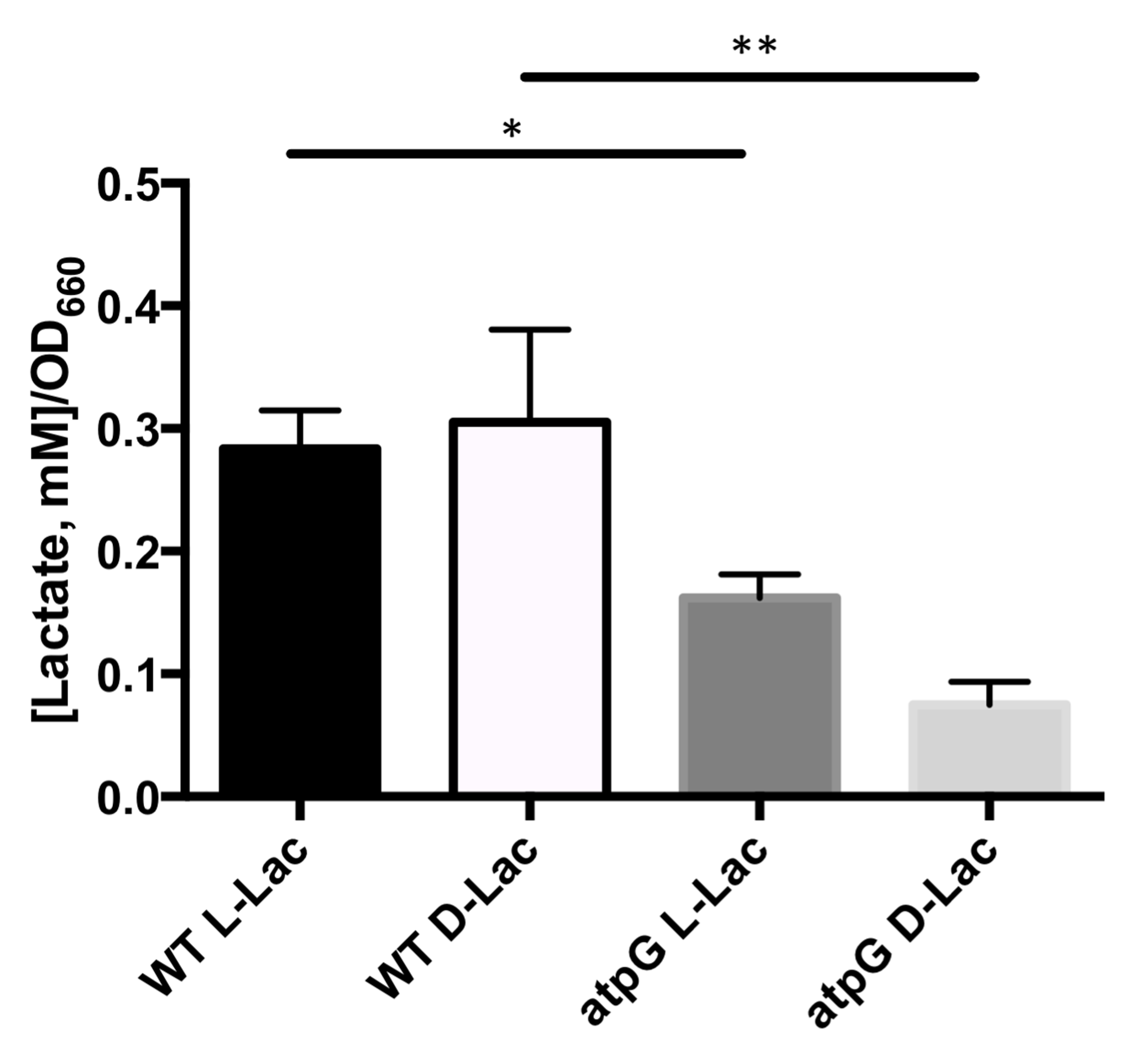

Supplement: S7 Fig — Both L- and D-lactate levels were determined before and 2-hr after NO· addition (10 mM DETA/NO) for both the WT and ∆atpG mutant and normalized to the change in OD650 over that same time period. The mutant consistently excreted 50% of the L-lactate and 33% of the D-lactate normally secreted by WT. Statistical significance was determined using Student’s t-test (n = 3, * p ≤ 0.05, ** p ≤ 0.01). (TIF) [file ppat.1006907.s007.tif]
